# Supplementary material for: Copper-Catalyzed One-Pot Synthesis of N-Sulfonyl Amidines from Sulfonyl Hydrazine, Terminal Alkynes and Sulfonyl Azides
Source: Molecules. 2021 Jun 17;26(12):3700. doi: 10.3390/molecules26123700 (PMC8235413; doi:10.3390/molecules26123700)
Supplement: Supplementary file 1 [file molecules-26-03700-s001.zip › molecules-1253738-supplementary.pdf]

Copper-catalyzed one-pot synthesis of *N*-sulfonyl amidines from sulfonyl hydrazine, 1-alkynes, and *N*-sulfonyl azides

Yu Zhao [#,1], Zitong Zhou [#,1], Man Chen [1] and Weiguang Yang [1,2,*]

[#] *These authors contributed equally*

[1] *The Marine Biomedical Research Institute, Guangdong Medical University, Zhanjiang, 524023, China.*

[2] *The Marine Biomedical Research Institute of Guangdong Zhanjiang, Zhanjiang, Guangdong, 524023, China.*

*Email: 09ywg@163.com

Table of contents

1. The structures of starting materials **1a-1f** 2

2. General Information 3

3 NMR spetra 4

1. The structures of starting materials 1a-1f

Structures of the starting materials 1a-1f

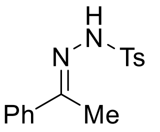

1a

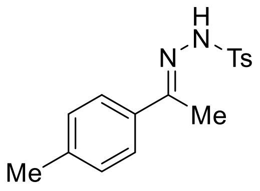

1b

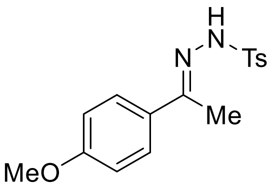

1c

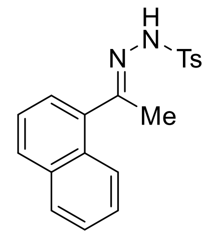

1d

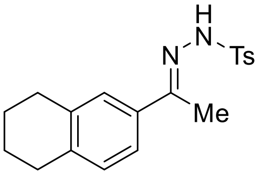

1e

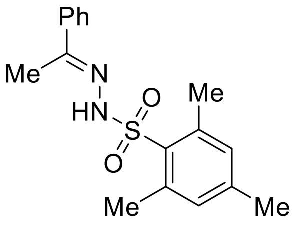

1f

Structures of the starting materials 2a-2f

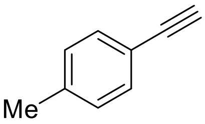

2a

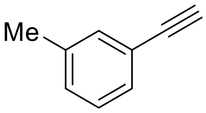

2b

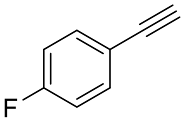

2c

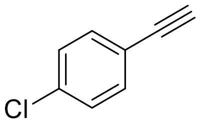

2d

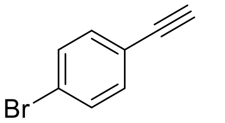

2e

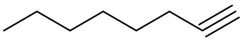

2f

Structures of the starting materials 3a-3e

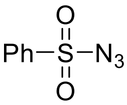

3a

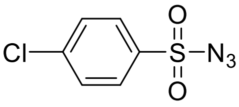

3b

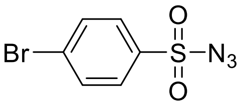

3c

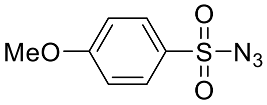

3d

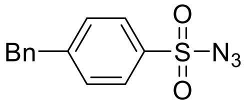

3e

2. General Information

All melting points were determined on a Yanaco melting point apparatus and were uncorrected. IR spectra were recorded as KBr pellets on a Nicolet FT-IR 5DX spectrometer. All spectra of ^{1}H NMR (400 MHz) and ^{13}C NMR (100 MHz) were measured on a 400 MHz Bruker spectrometer using DMSO-d_6 or $CDCl_3$ as the solvent with tetramethylsilane (TMS) as the internal standard at room temperature. . Chemical shifts are given in δ relative to TMS, the coupling constants *J* are given in Hz. HRMS were obtained on a Bruker micrOTOF-Q II spectrometer.

3 NMR spetra

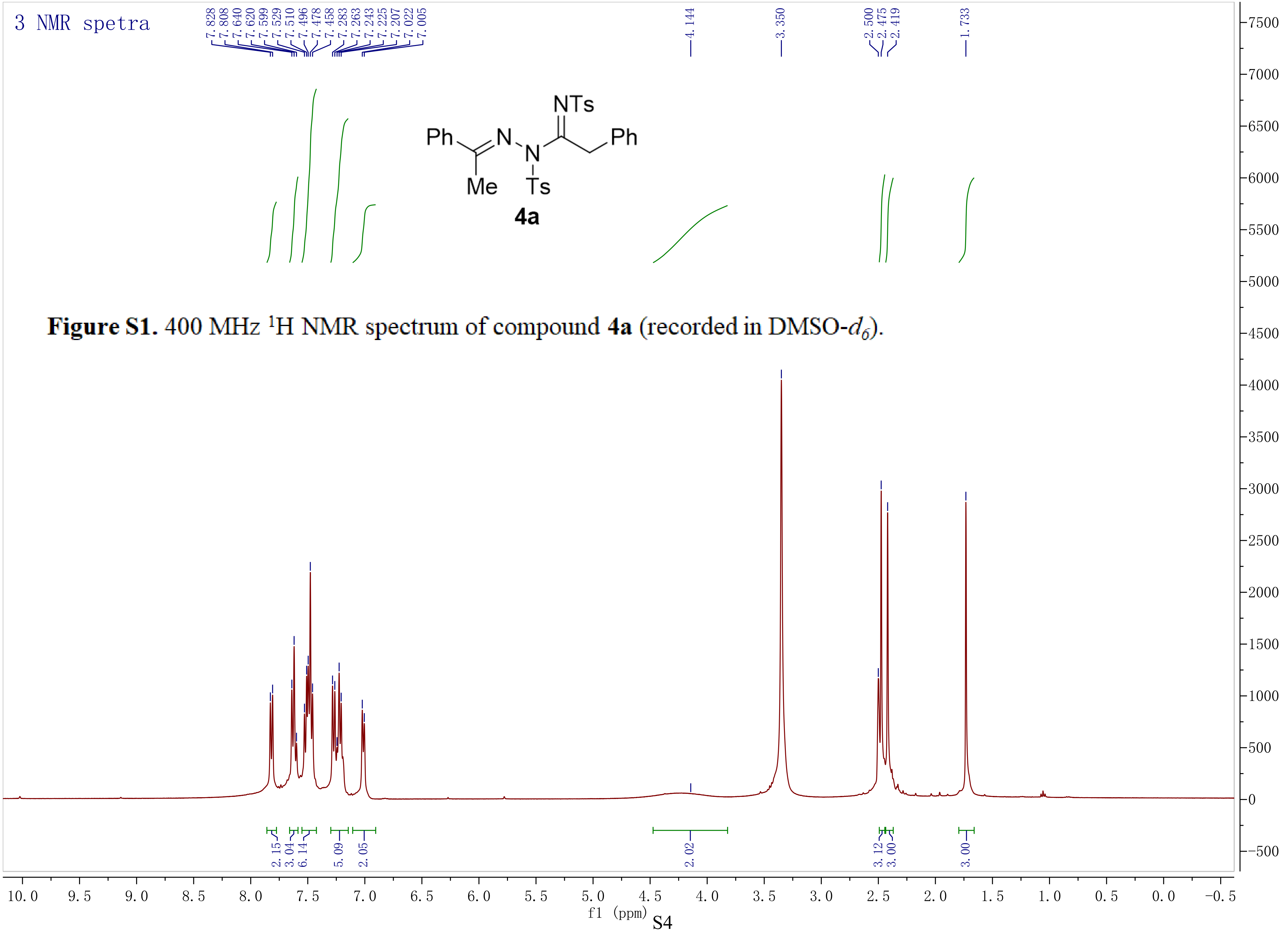

Figure S1. 400 MHz ^{1}H NMR spectrum of compound **4a** (recorded in DMSO-d_6).

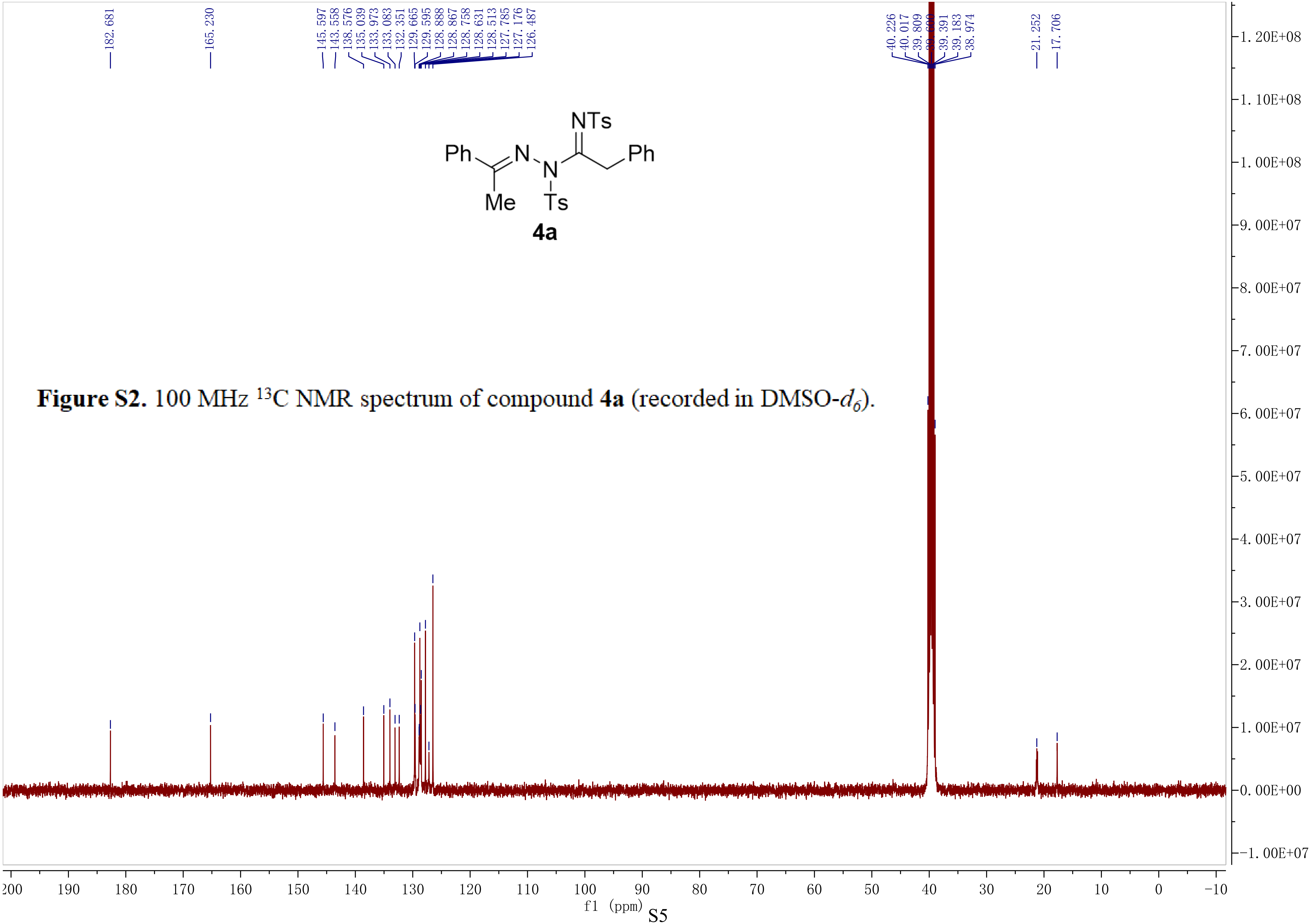

Figure S2. 100 MHz ^{13}C NMR spectrum of compound **4a** (recorded in DMSO-d_6).

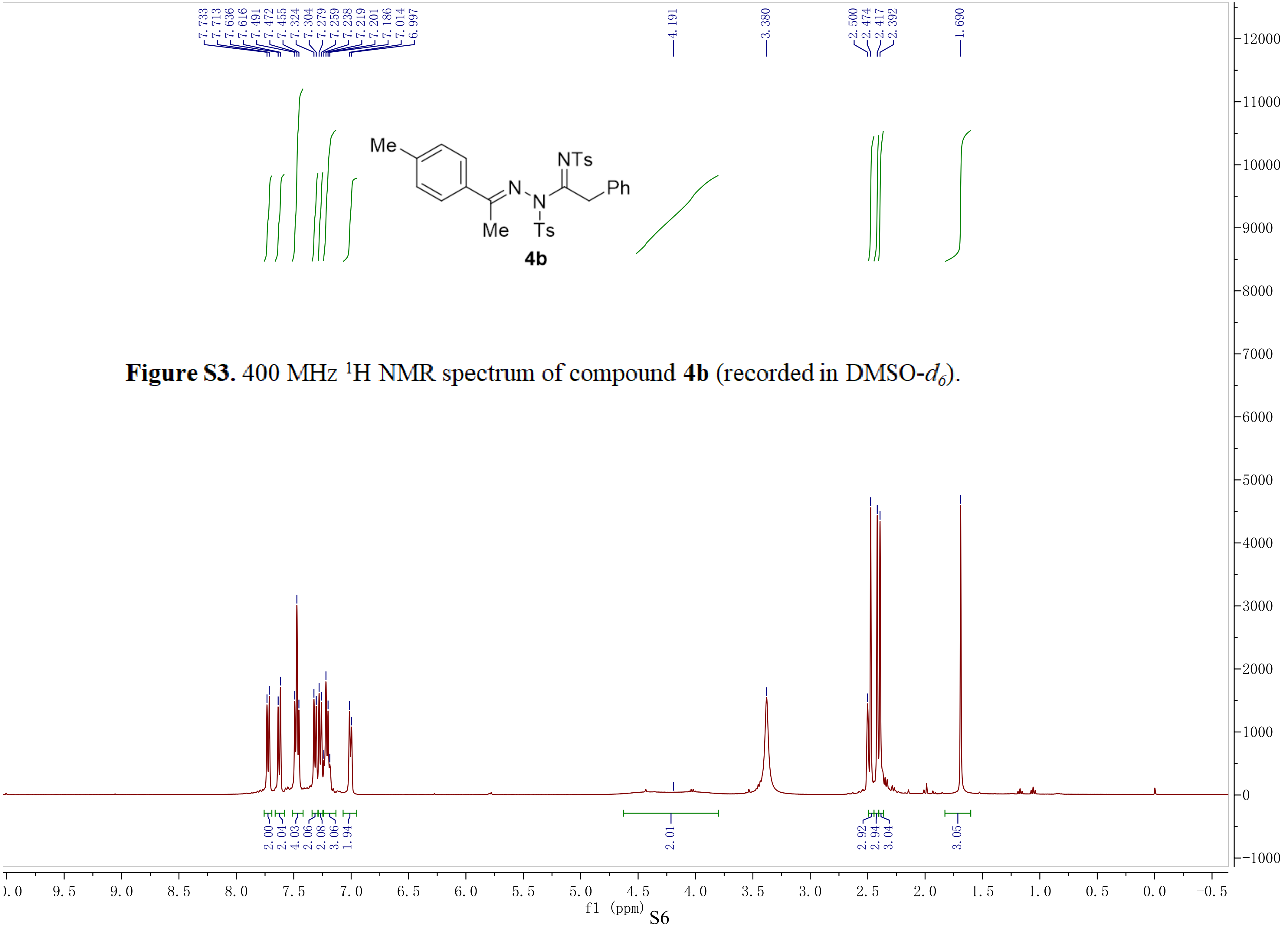

Figure S3. 400 MHz ^{1}H NMR spectrum of compound **4b** (recorded in DMSO-d_6).

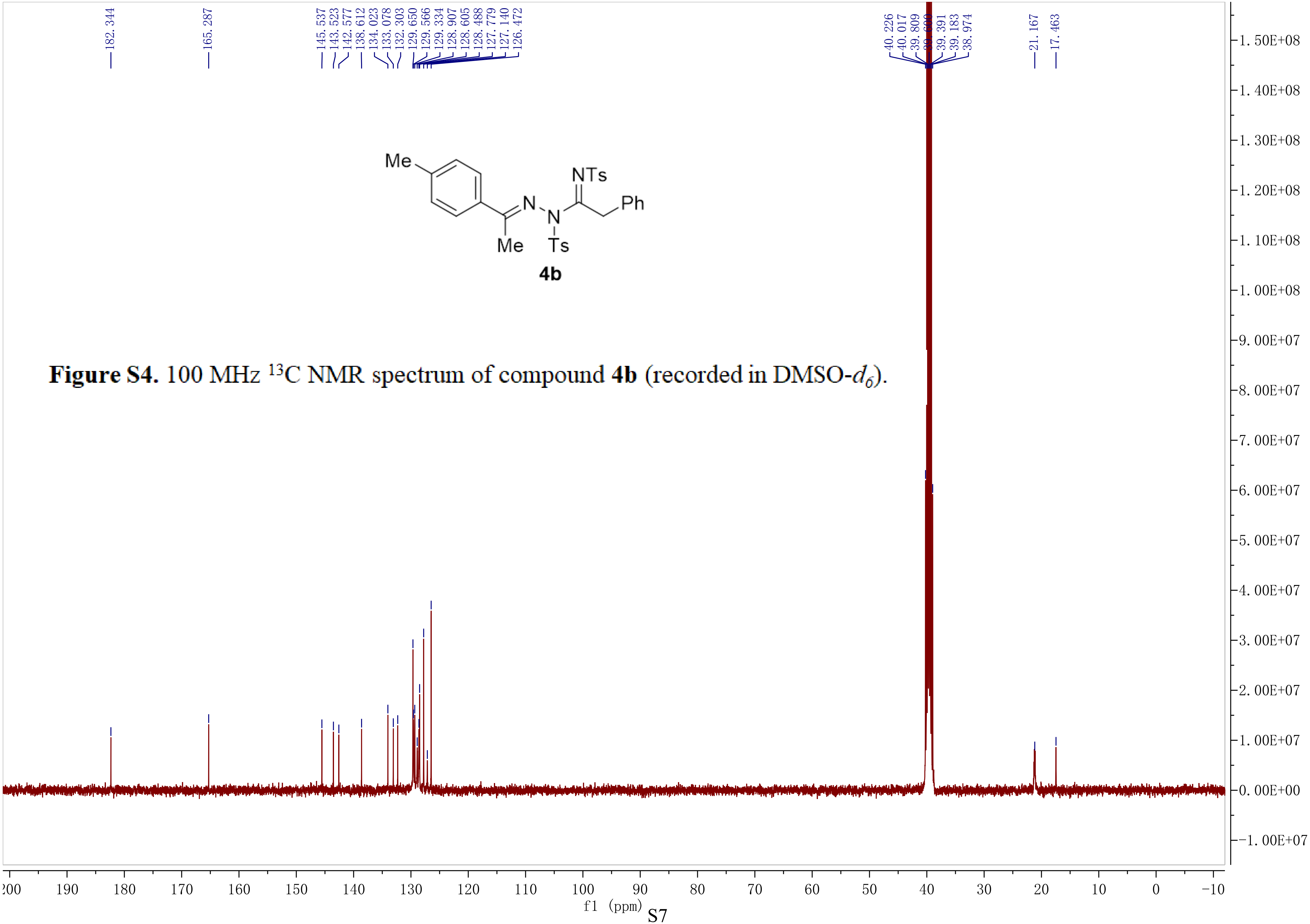

Figure S4. 100 MHz ^{13}C NMR spectrum of compound **4b** (recorded in DMSO-d_6).

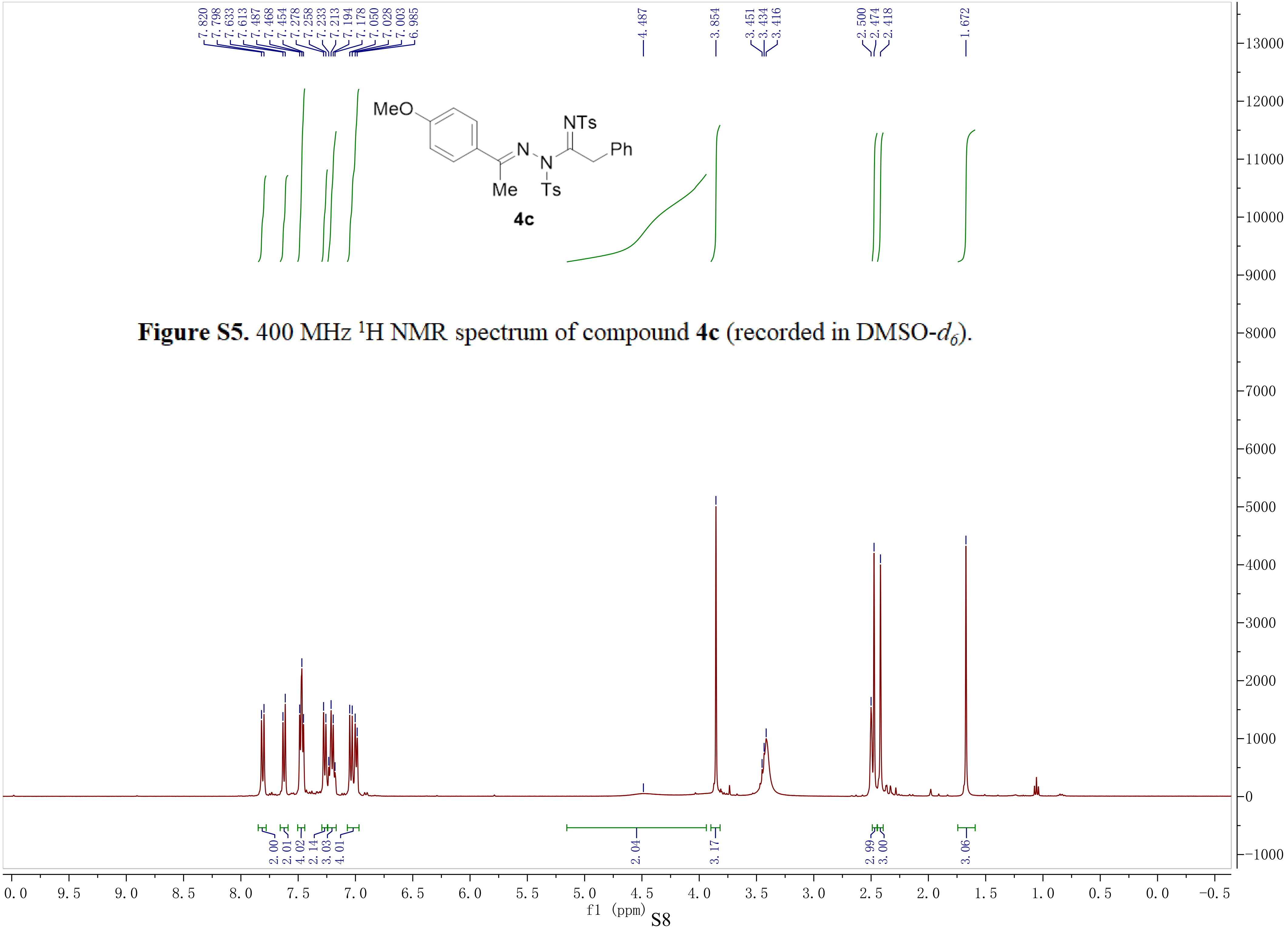

Figure S5. 400 MHz ^{1}H NMR spectrum of compound **4c** (recorded in DMSO-d_6).

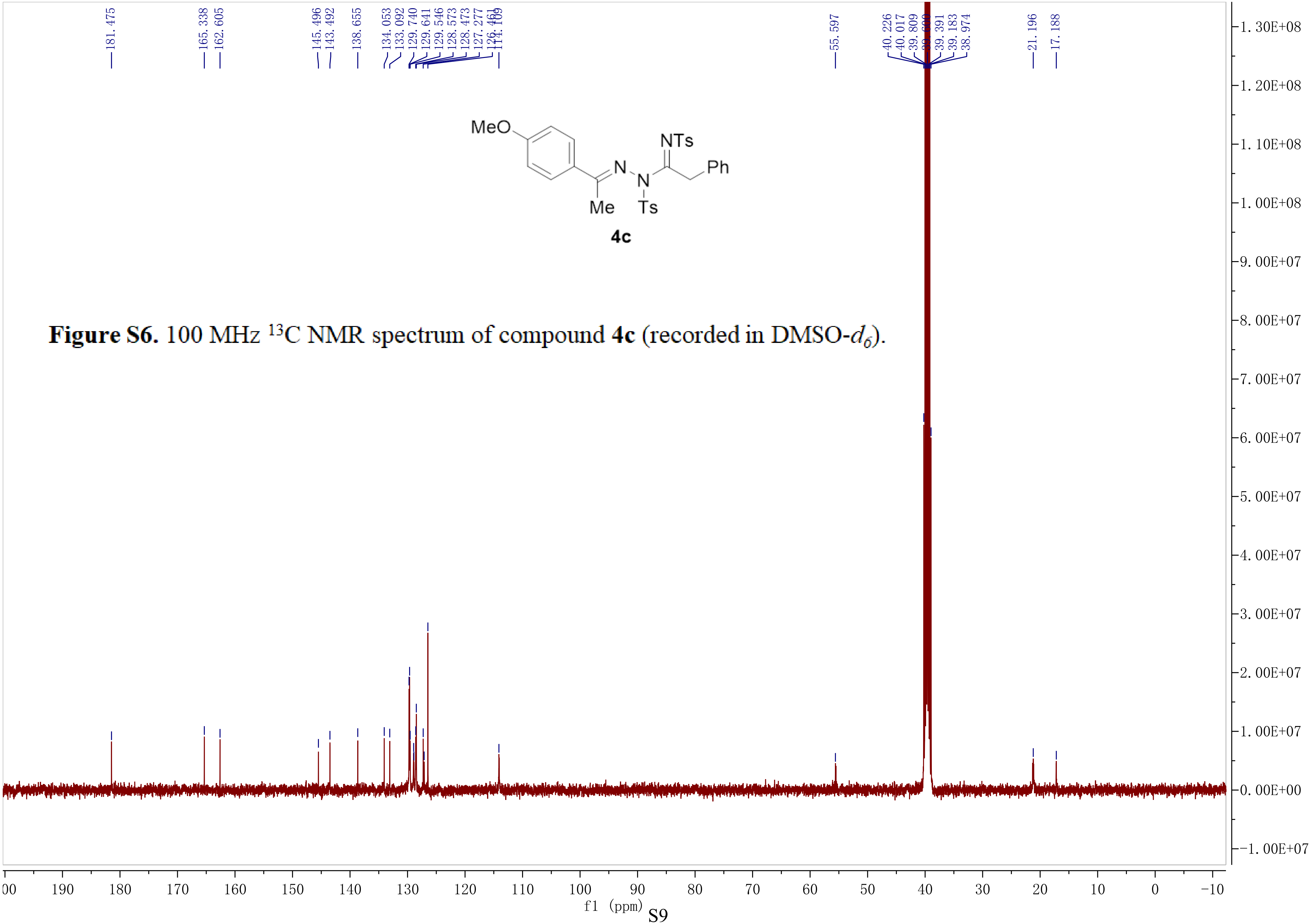

Figure S6. 100 MHz ^{13}C NMR spectrum of compound **4c** (recorded in DMSO-d_6).

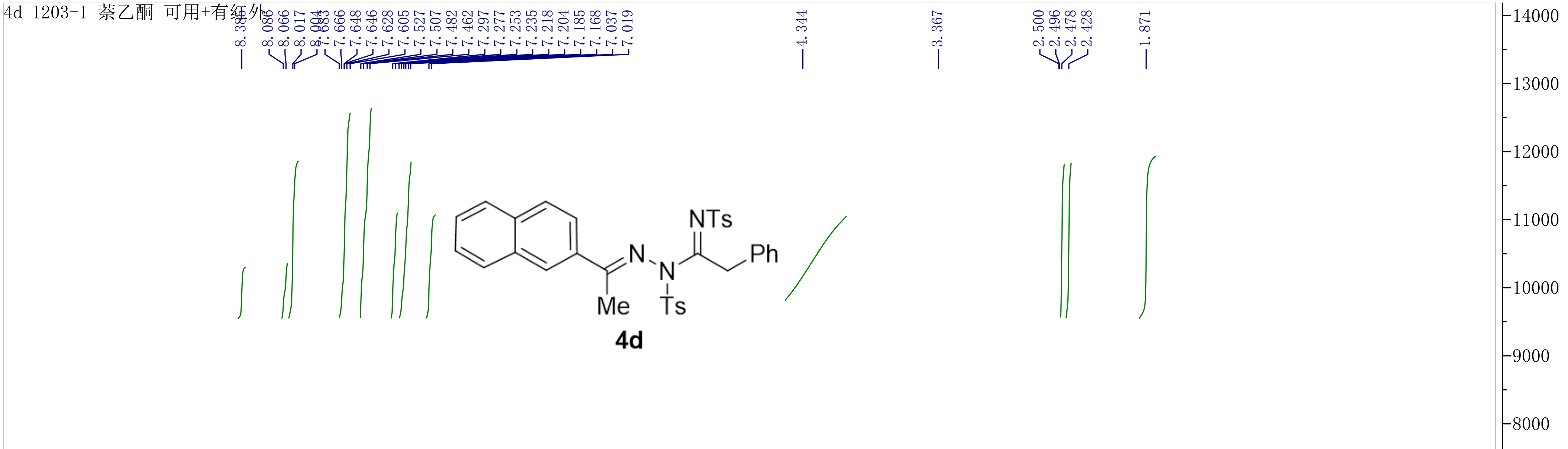

Figure S7. 400 MHz ^{1}H NMR spectrum of compound **4d** (recorded in DMSO-d_6).

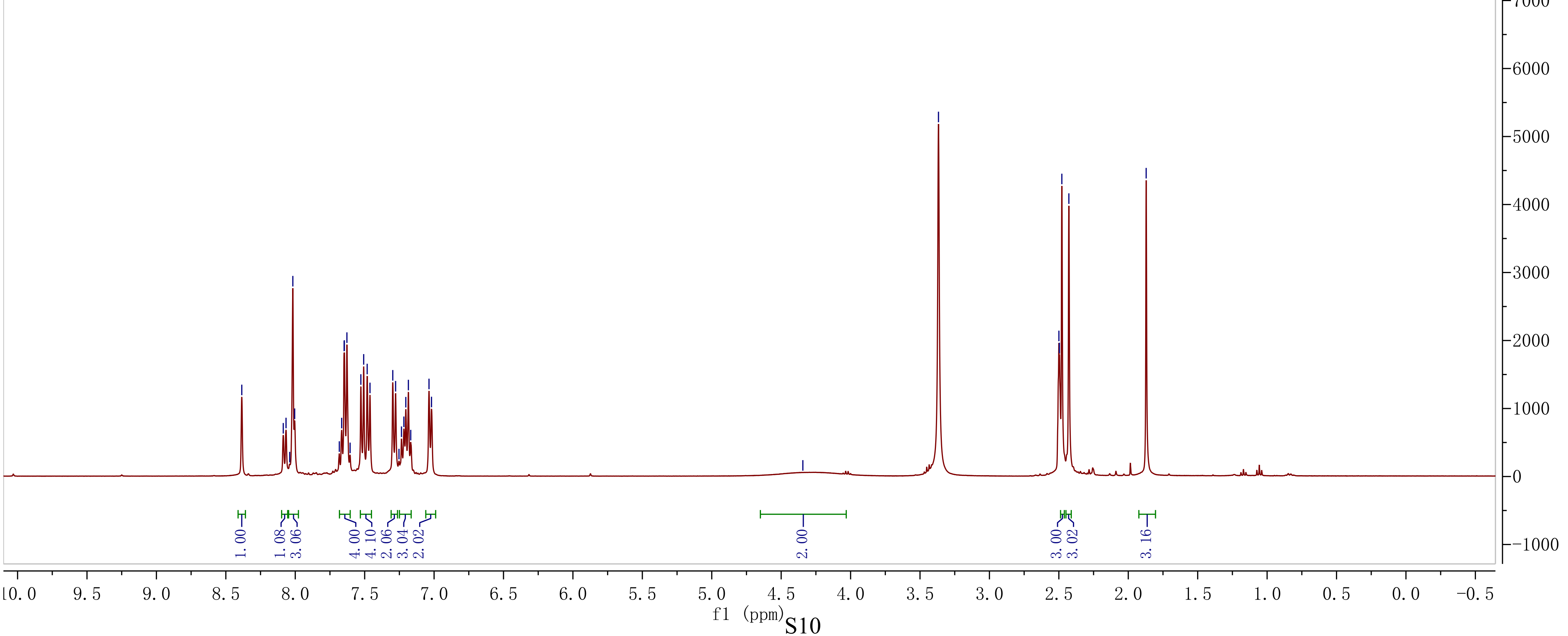

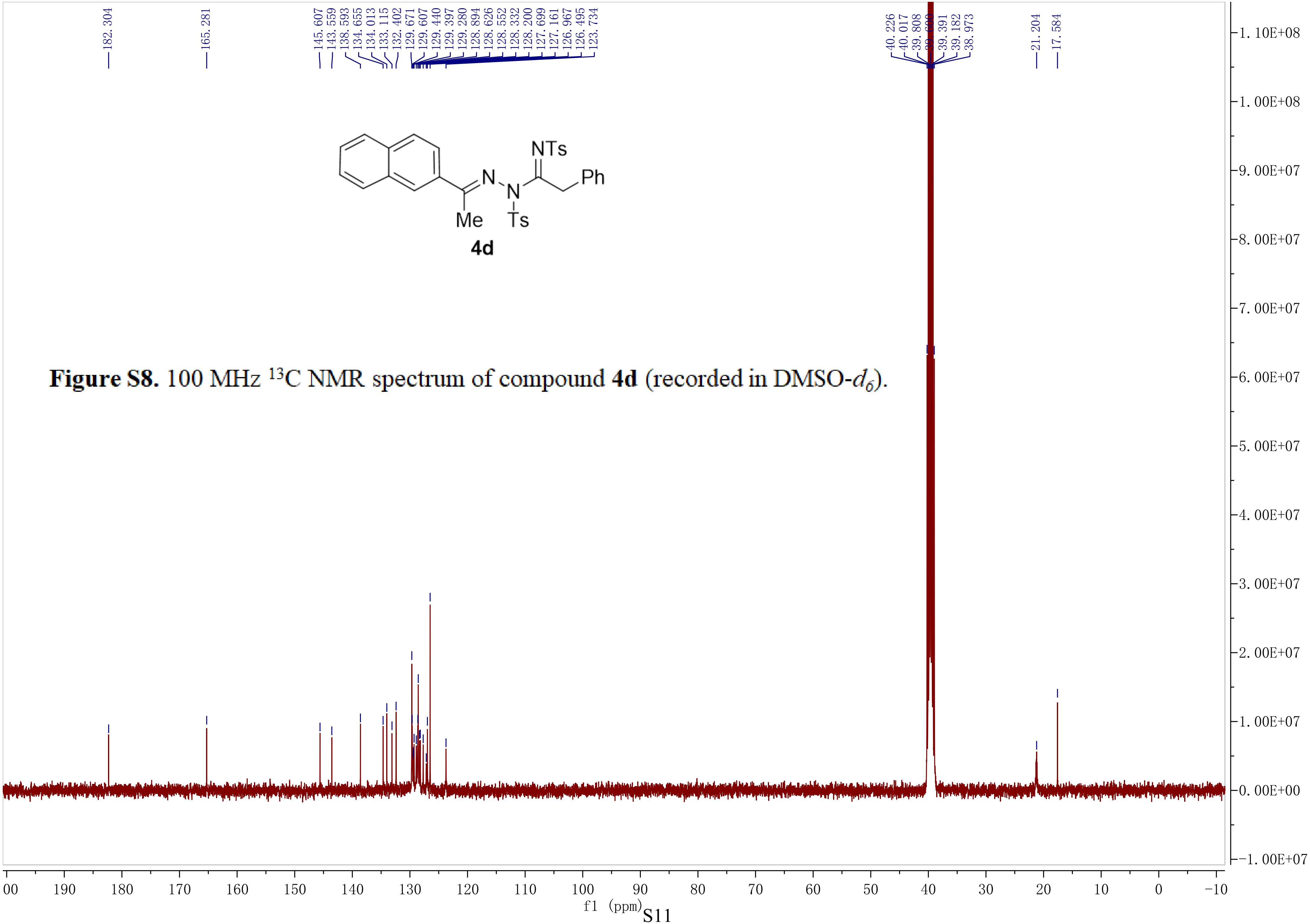

Figure S8. 100 MHz ^{13}C NMR spectrum of compound **4d** (recorded in DMSO-d_6).

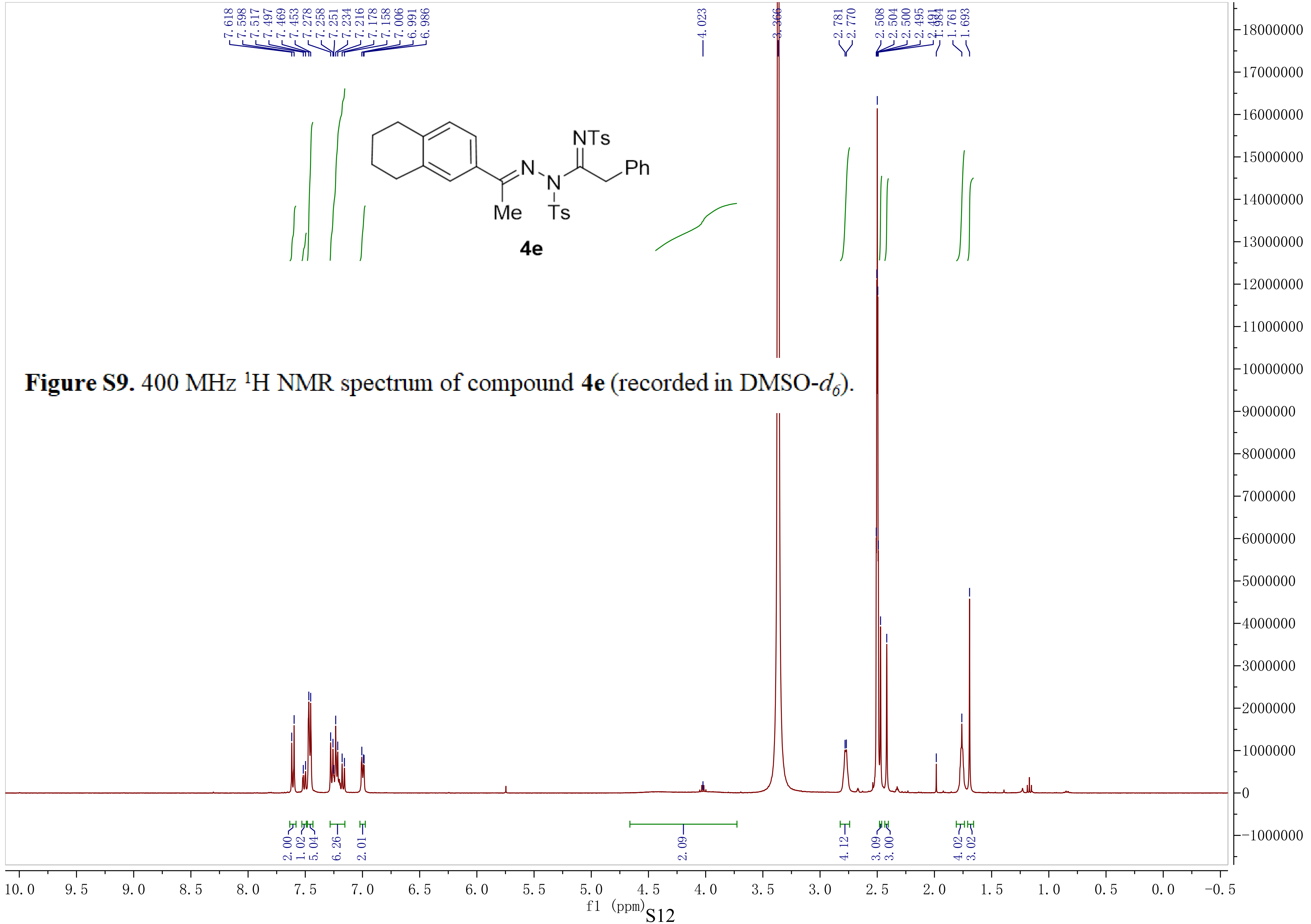

Figure S9. 400 MHz ^{1}H NMR spectrum of compound **4e** (recorded in DMSO-d_6).

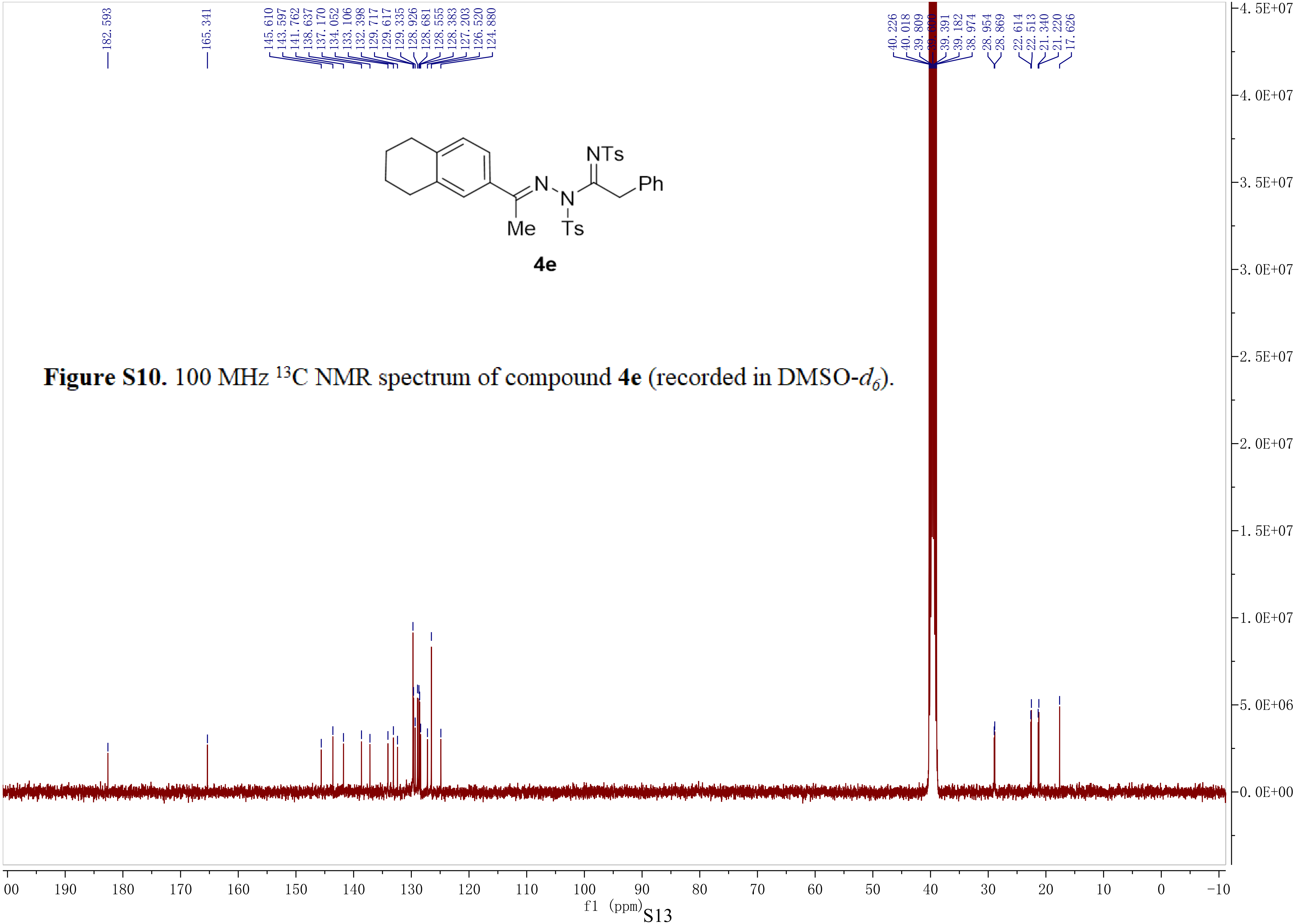

Figure S10. 100 MHz ^{13}C NMR spectrum of compound **4e** (recorded in DMSO-d_6).

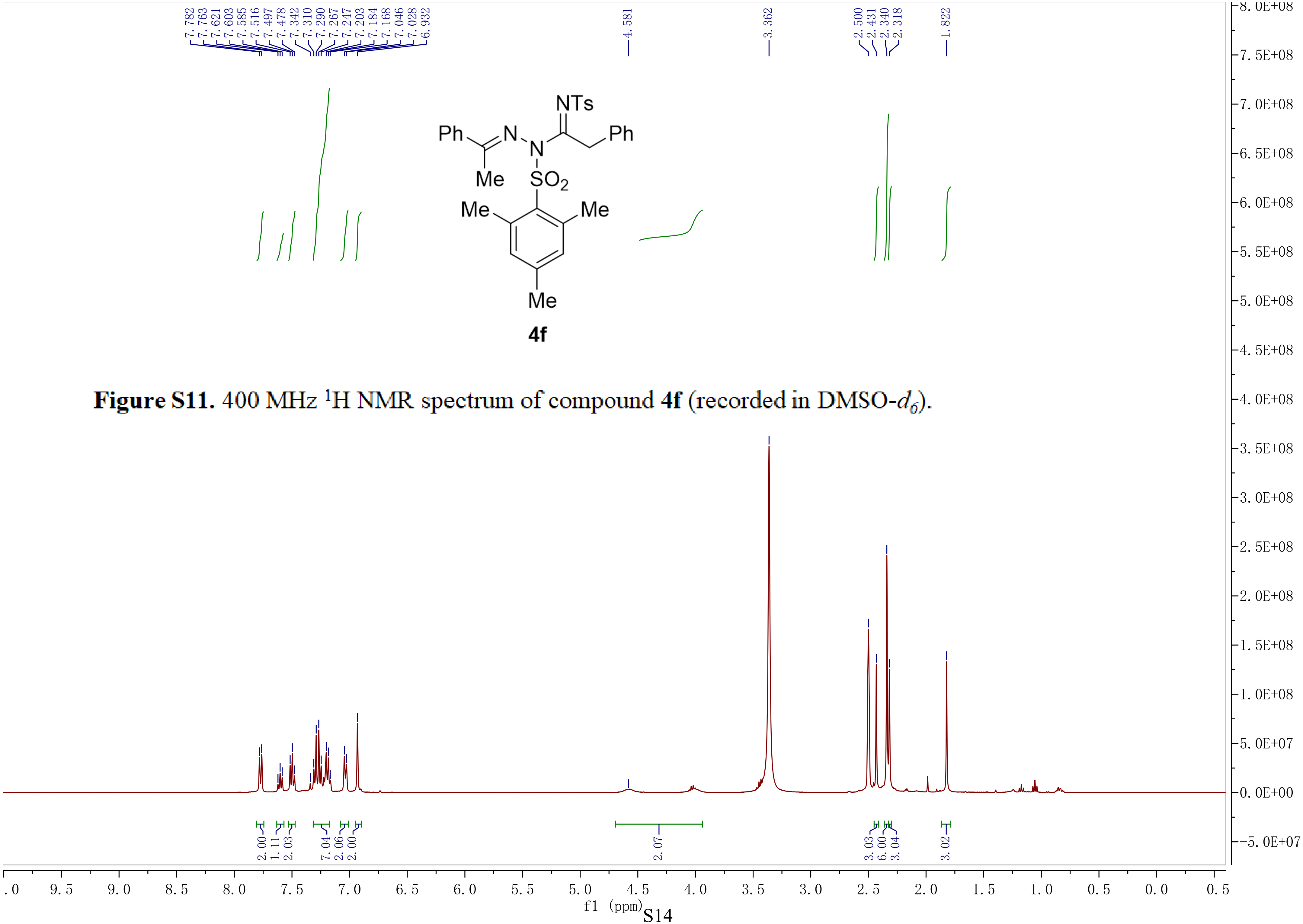

Figure S11. 400 MHz ^{1}H NMR spectrum of compound **4f** (recorded in DMSO-d_6).

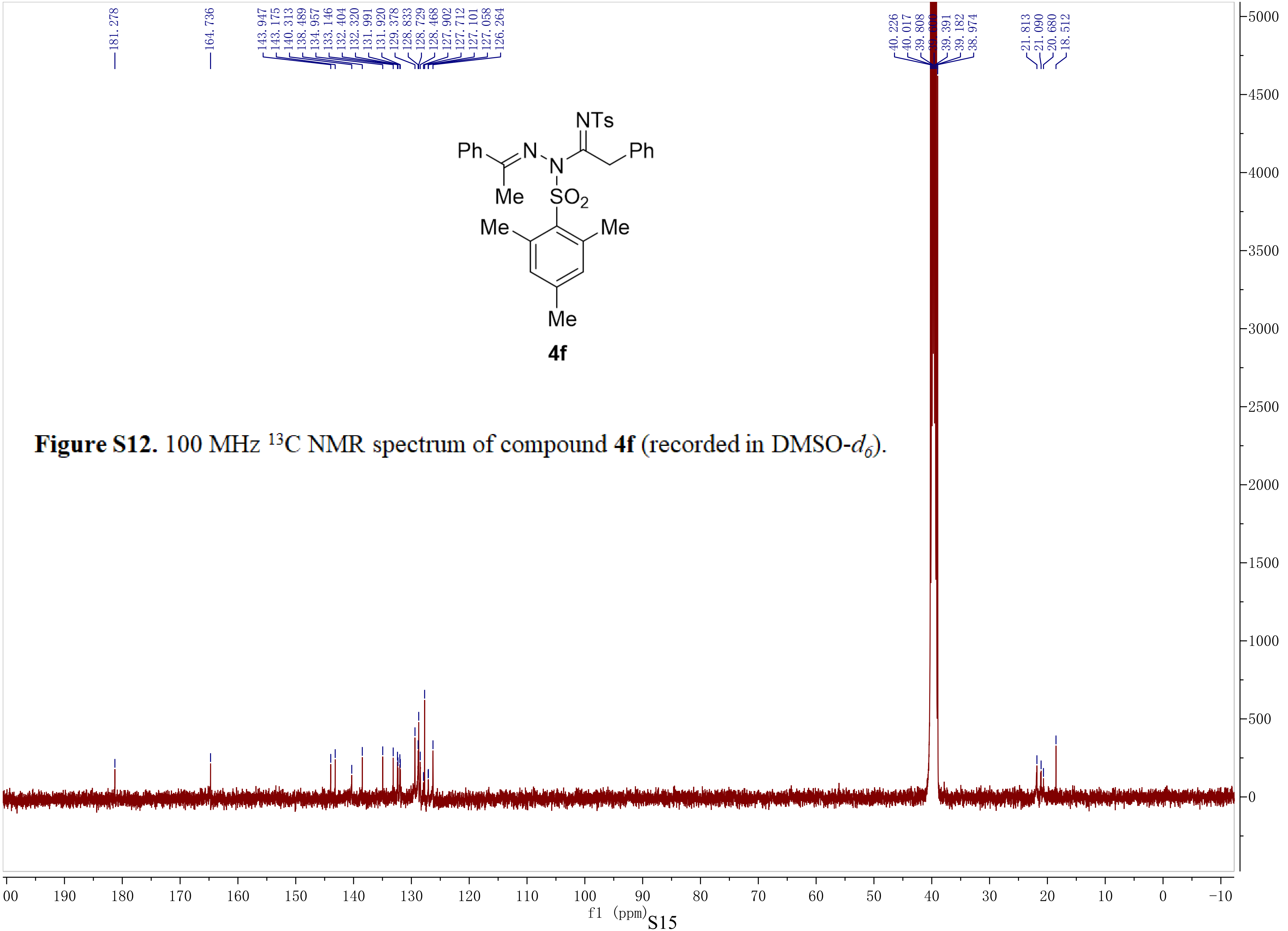

Figure S12. 100 MHz ^{13}C NMR spectrum of compound **4f** (recorded in DMSO-d_6).

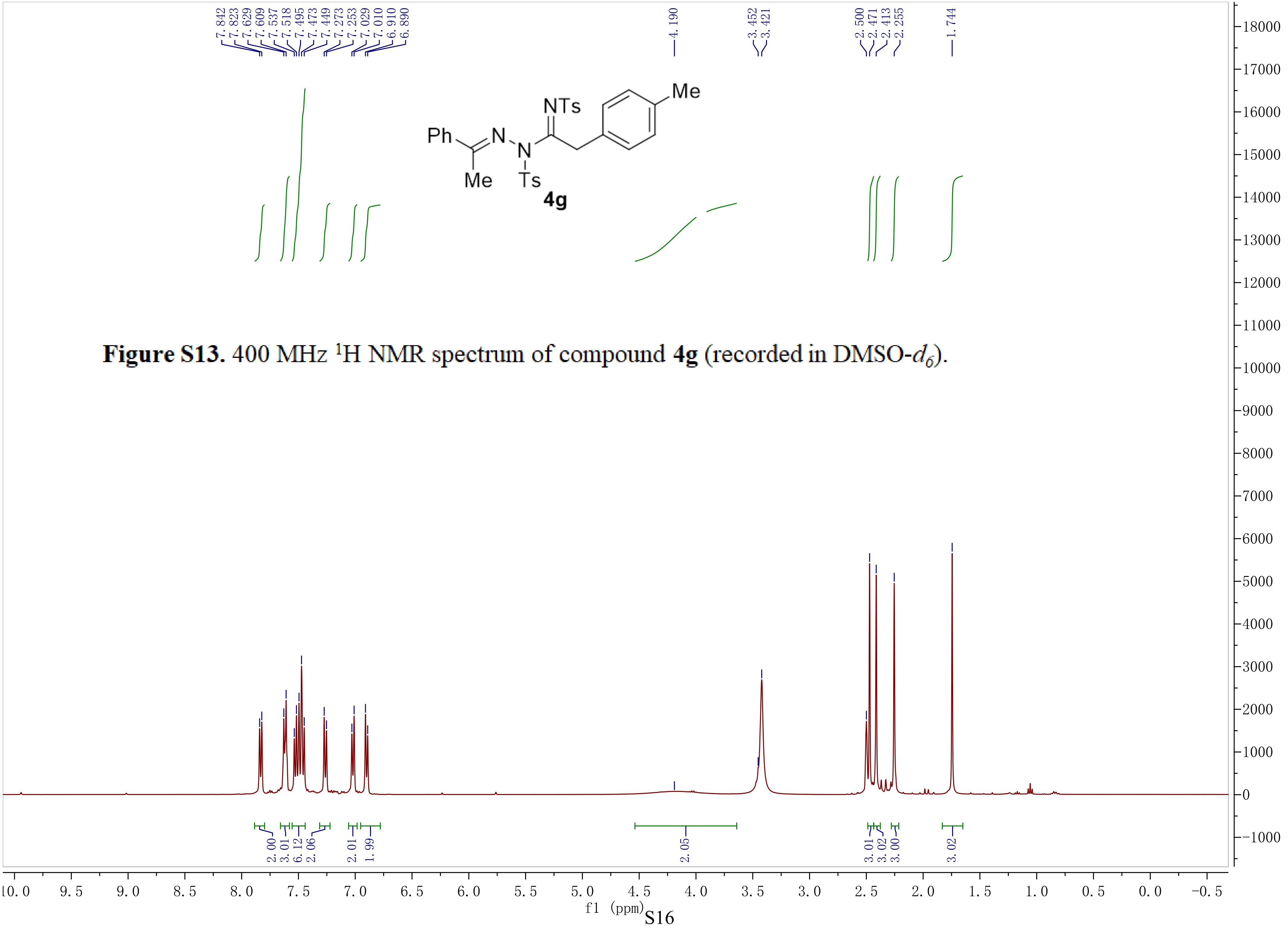

Figure S13. 400 MHz ^{1}H NMR spectrum of compound **4g** (recorded in DMSO-d_6).

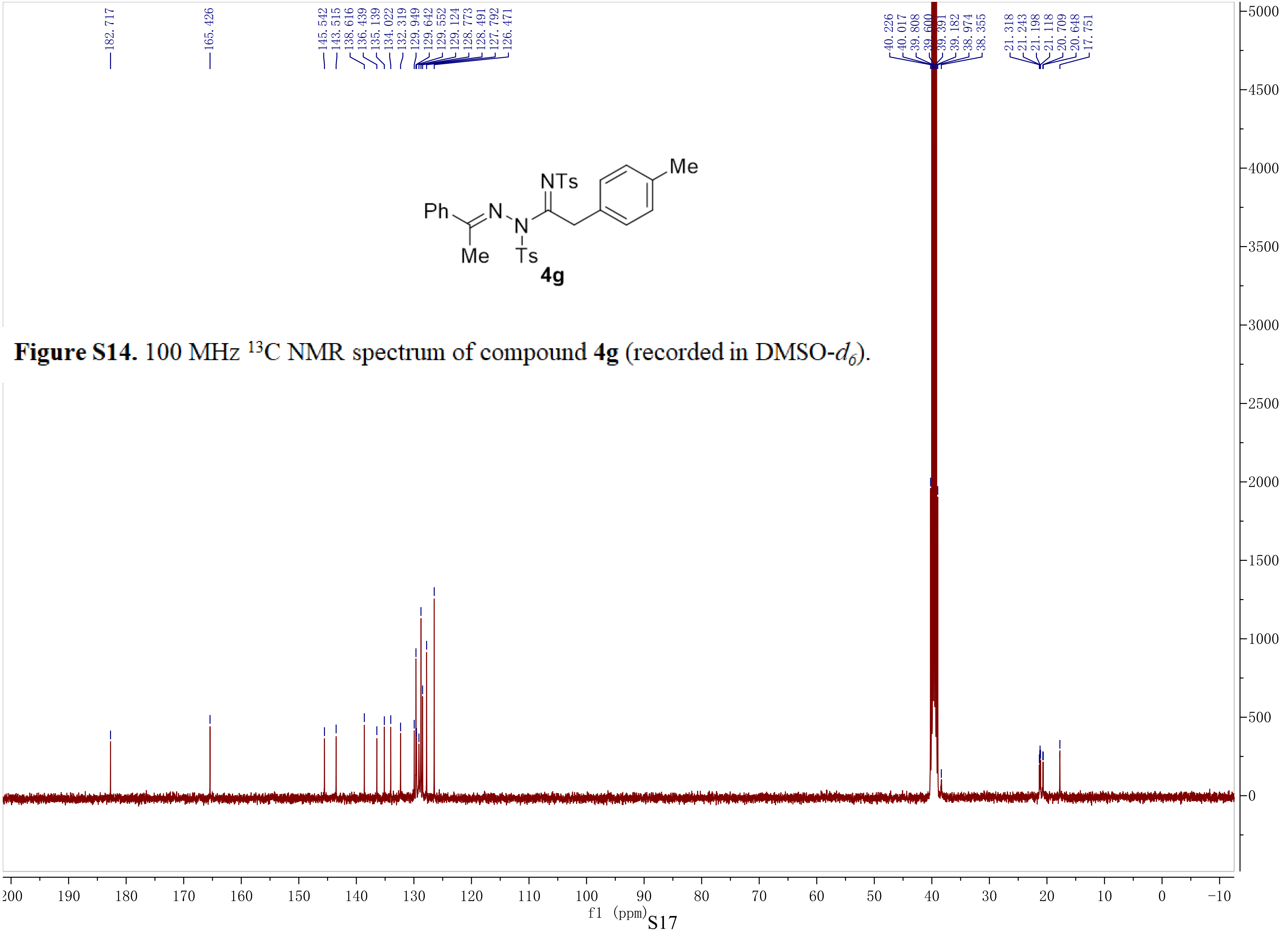

Figure S14. 100 MHz ^{13}C NMR spectrum of compound **4g** (recorded in DMSO-d_6).

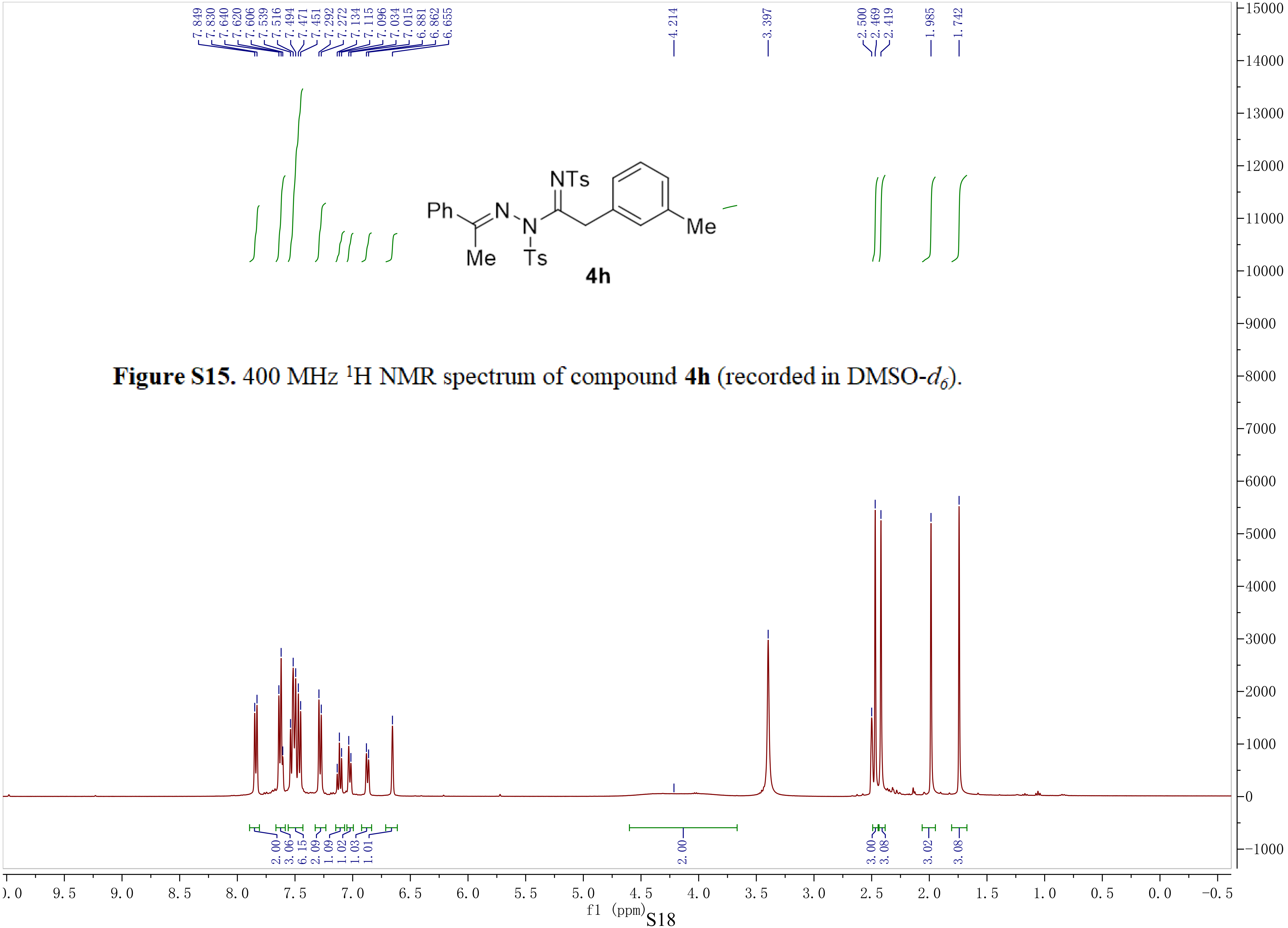

Figure S15. 400 MHz ^{1}H NMR spectrum of compound **4h** (recorded in DMSO-d_6).

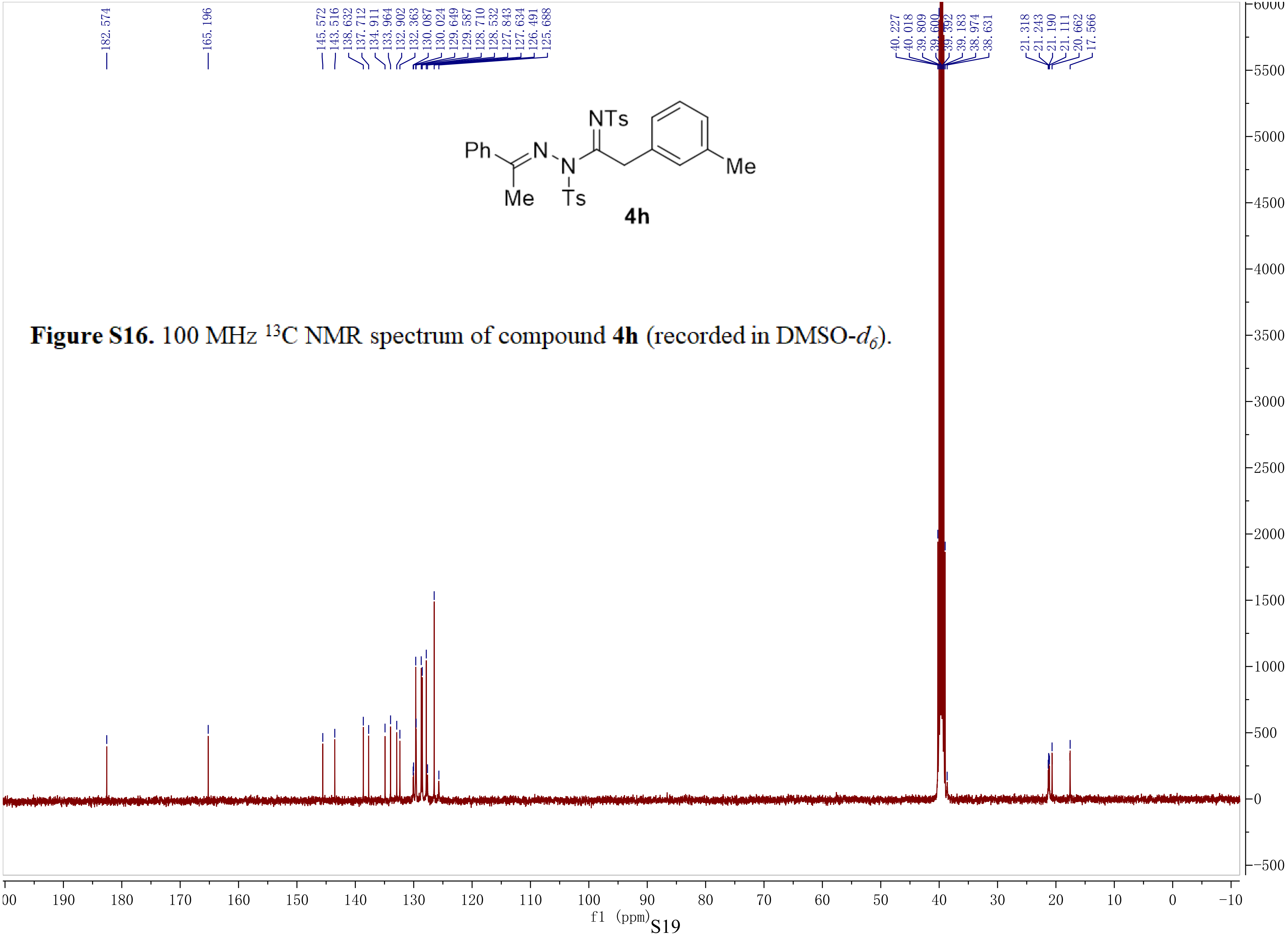

Figure S16. 100 MHz ^{13}C NMR spectrum of compound **4h** (recorded in DMSO-d_6).

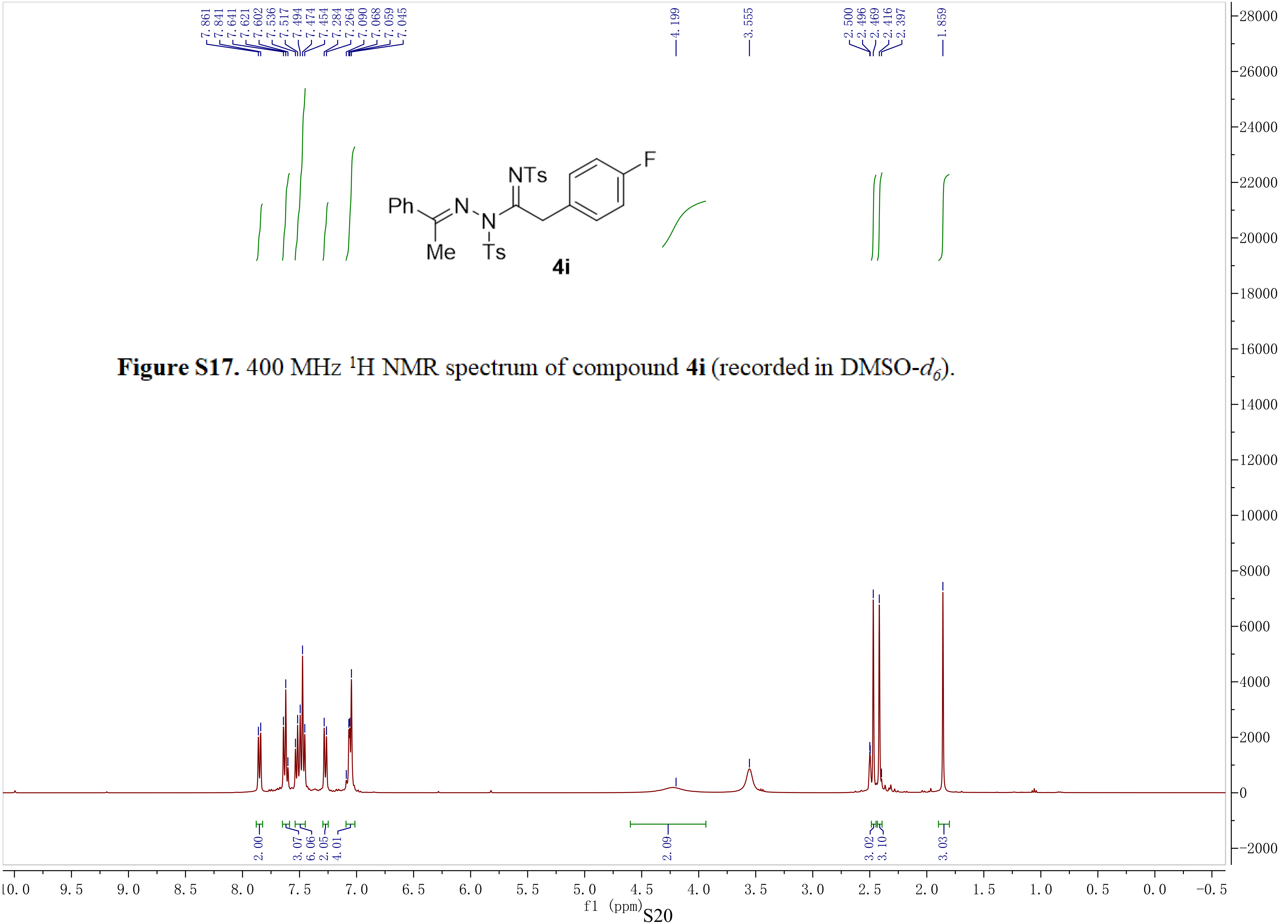

Figure S17. 400 MHz ^{1}H NMR spectrum of compound **4i** (recorded in DMSO-d_6).

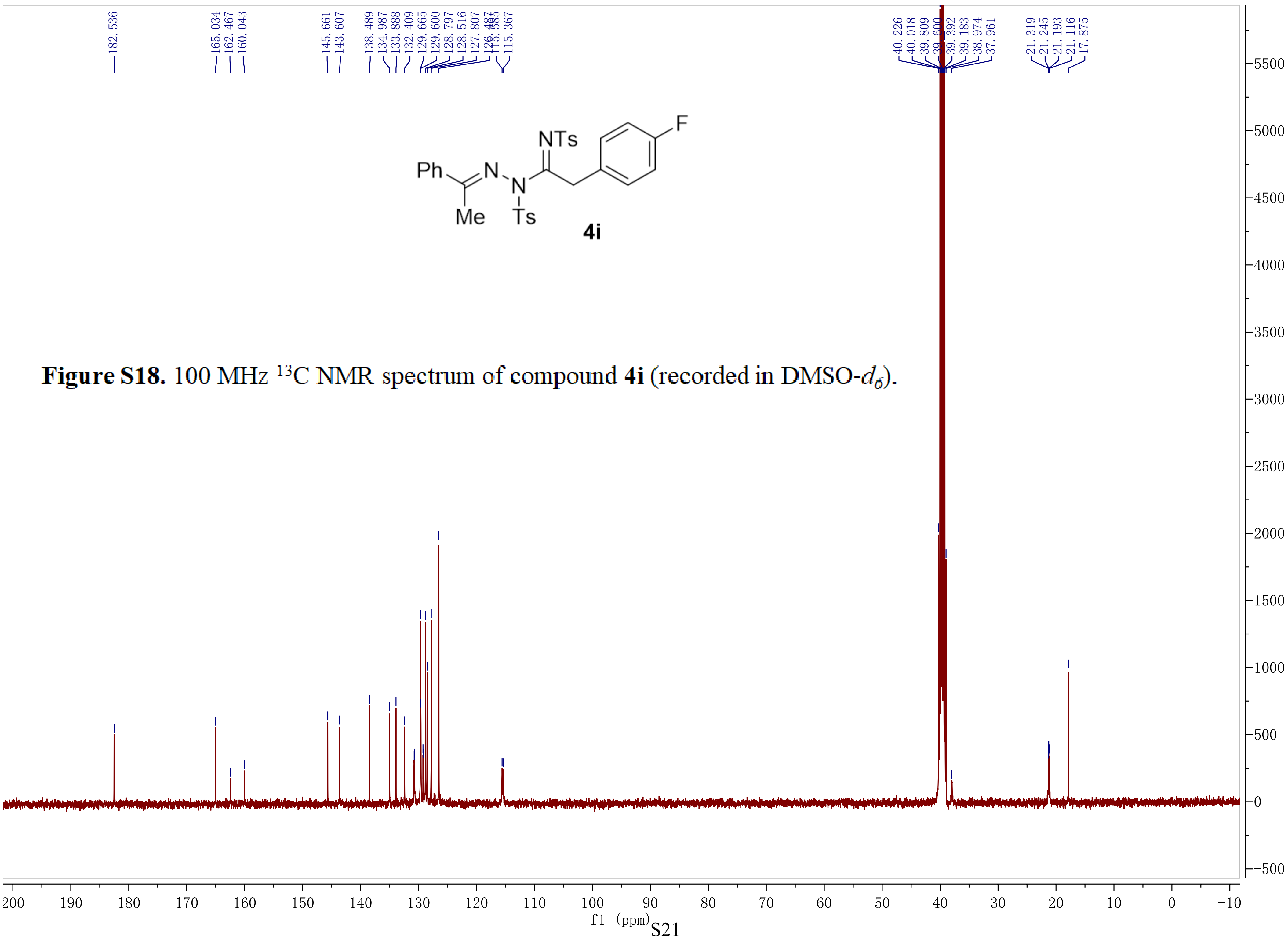

Figure S18. 100 MHz ^{13}C NMR spectrum of compound **4i** (recorded in DMSO-d_6).

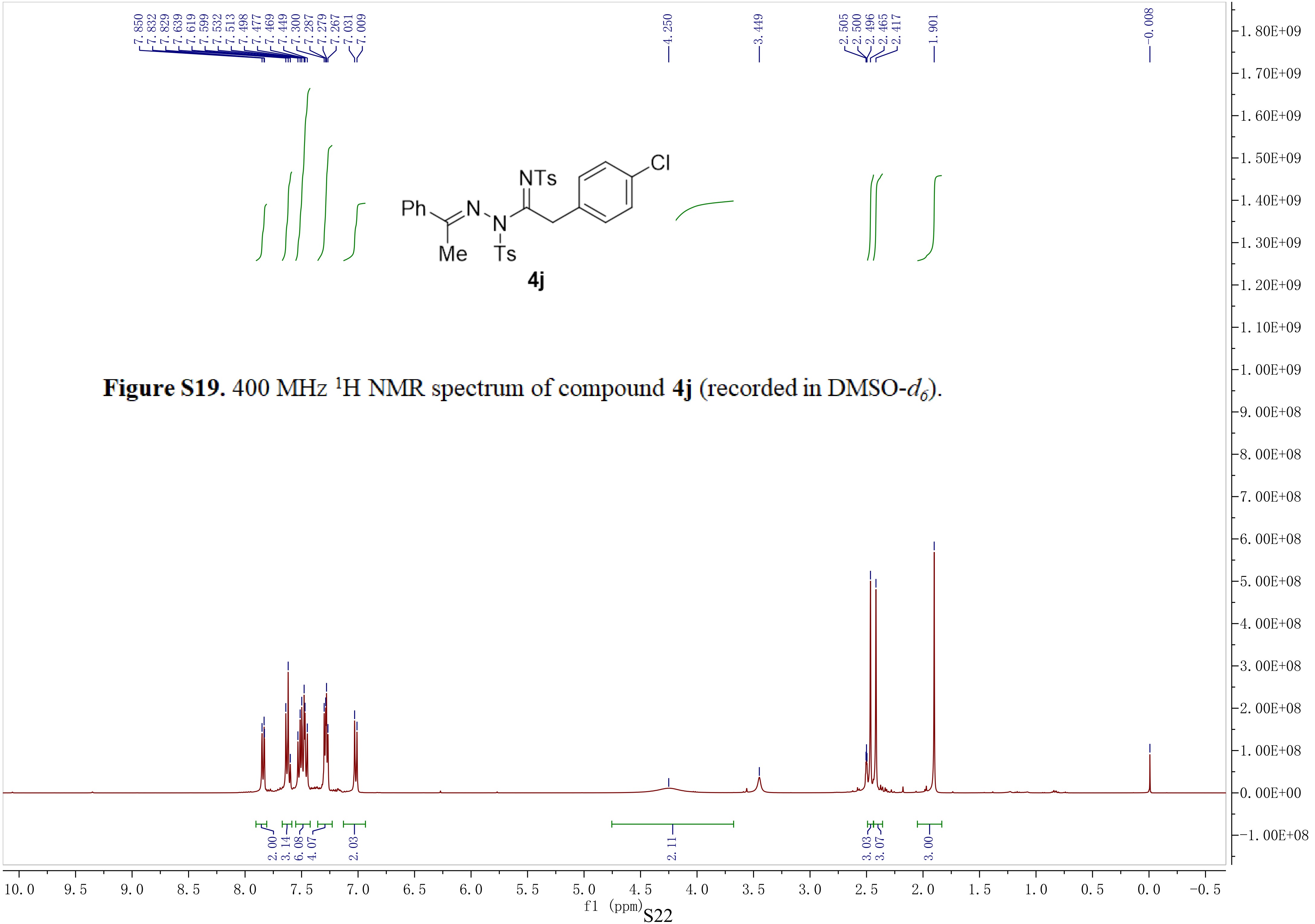

Figure S19. 400 MHz ^{1}H NMR spectrum of compound **4j** (recorded in DMSO-d_6).

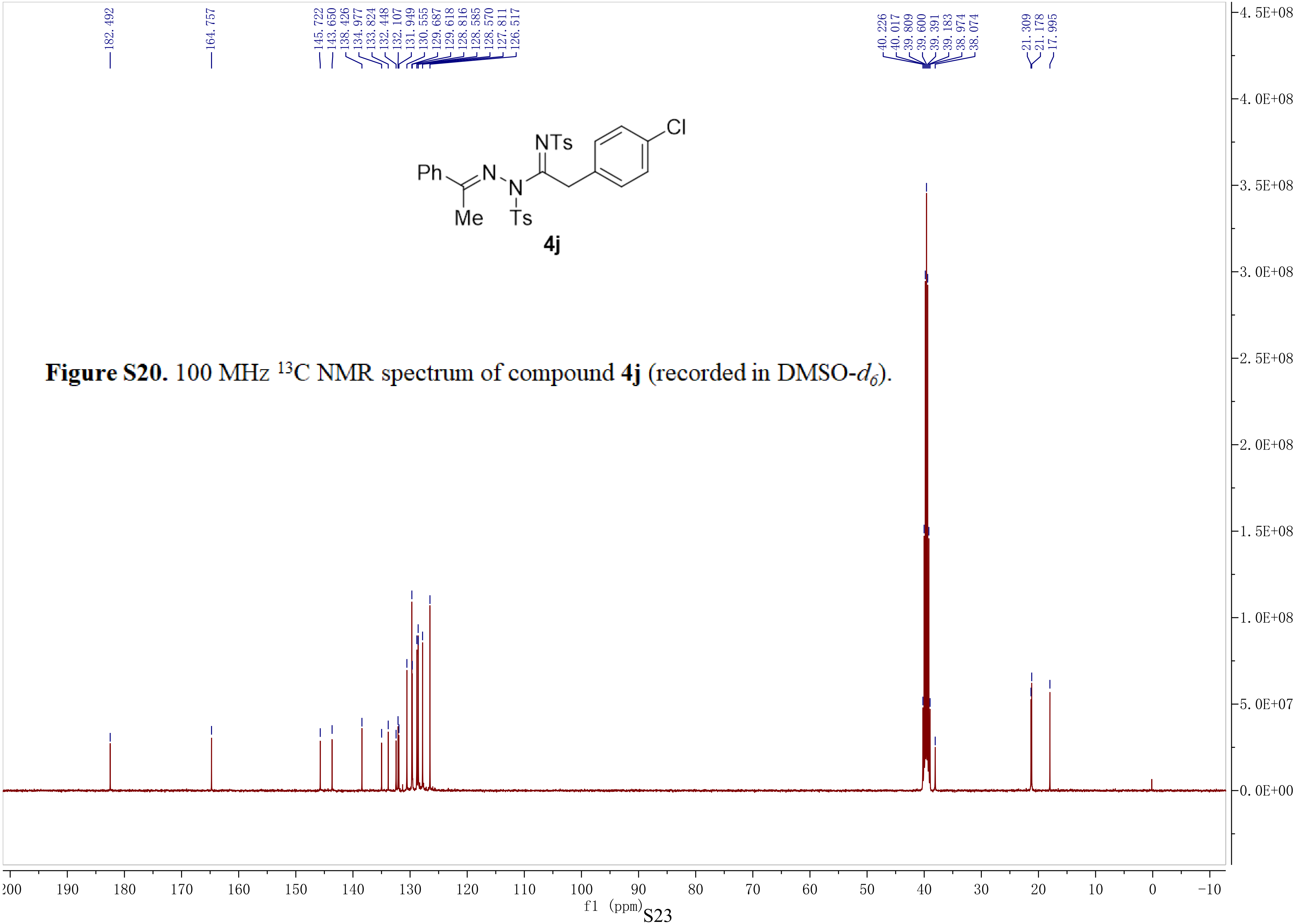

Figure S20. 100 MHz ^{13}C NMR spectrum of compound **4j** (recorded in DMSO-d_6).

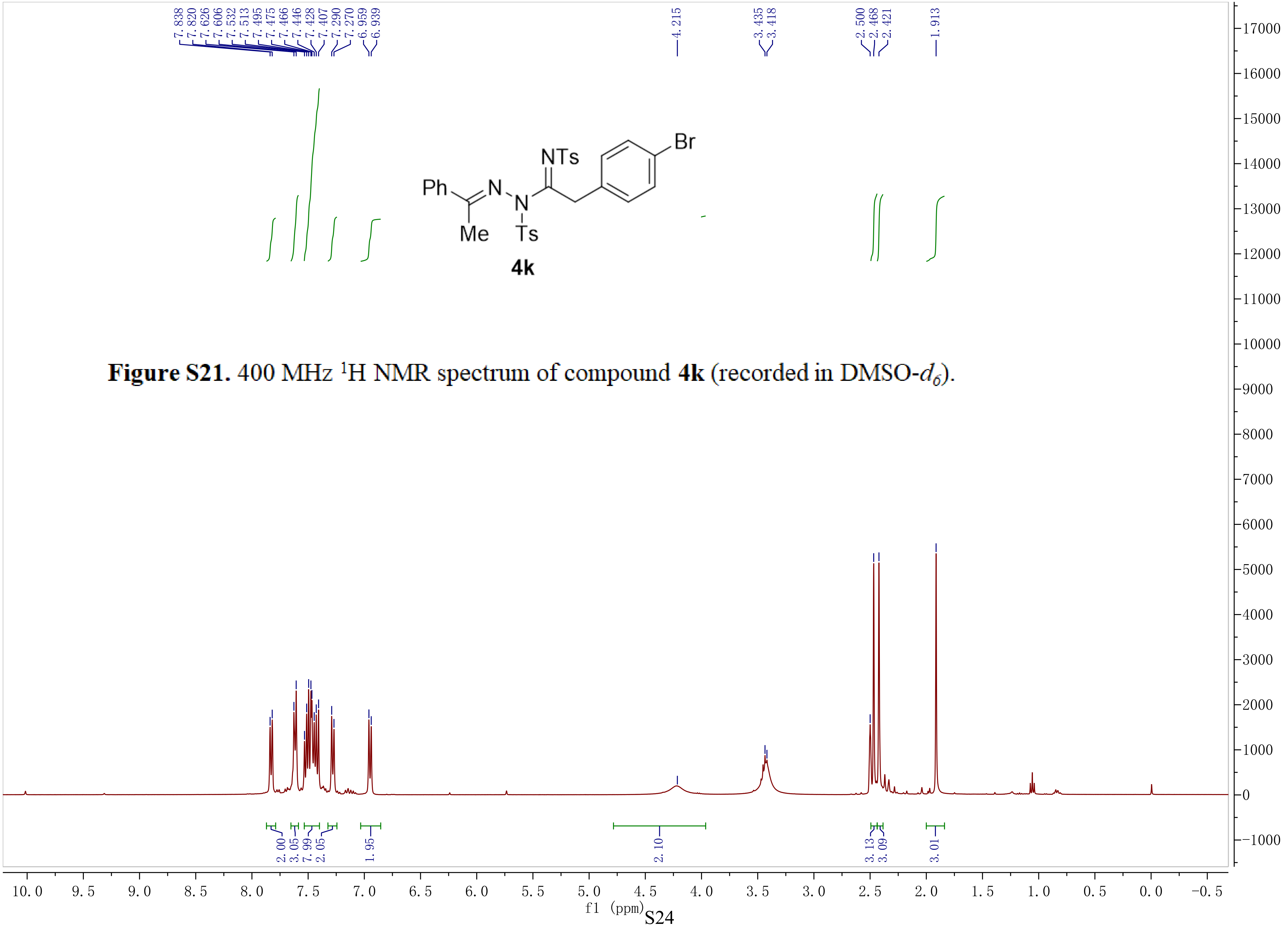

Figure S21. 400 MHz ¹H NMR spectrum of compound **4k** (recorded in DMSO-d_6).

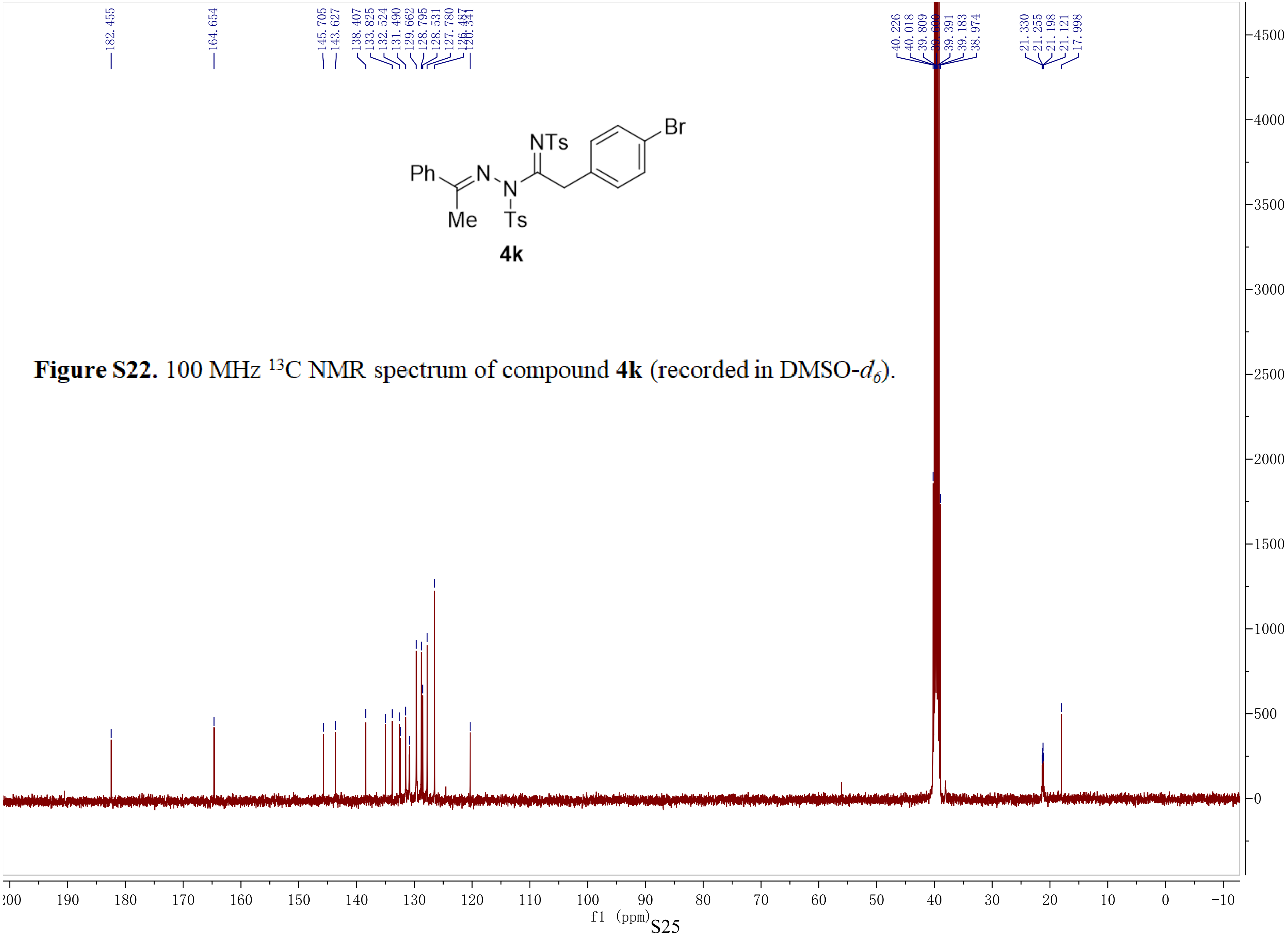

Figure S22. 100 MHz ^{13}C NMR spectrum of compound **4k** (recorded in DMSO-d_6).

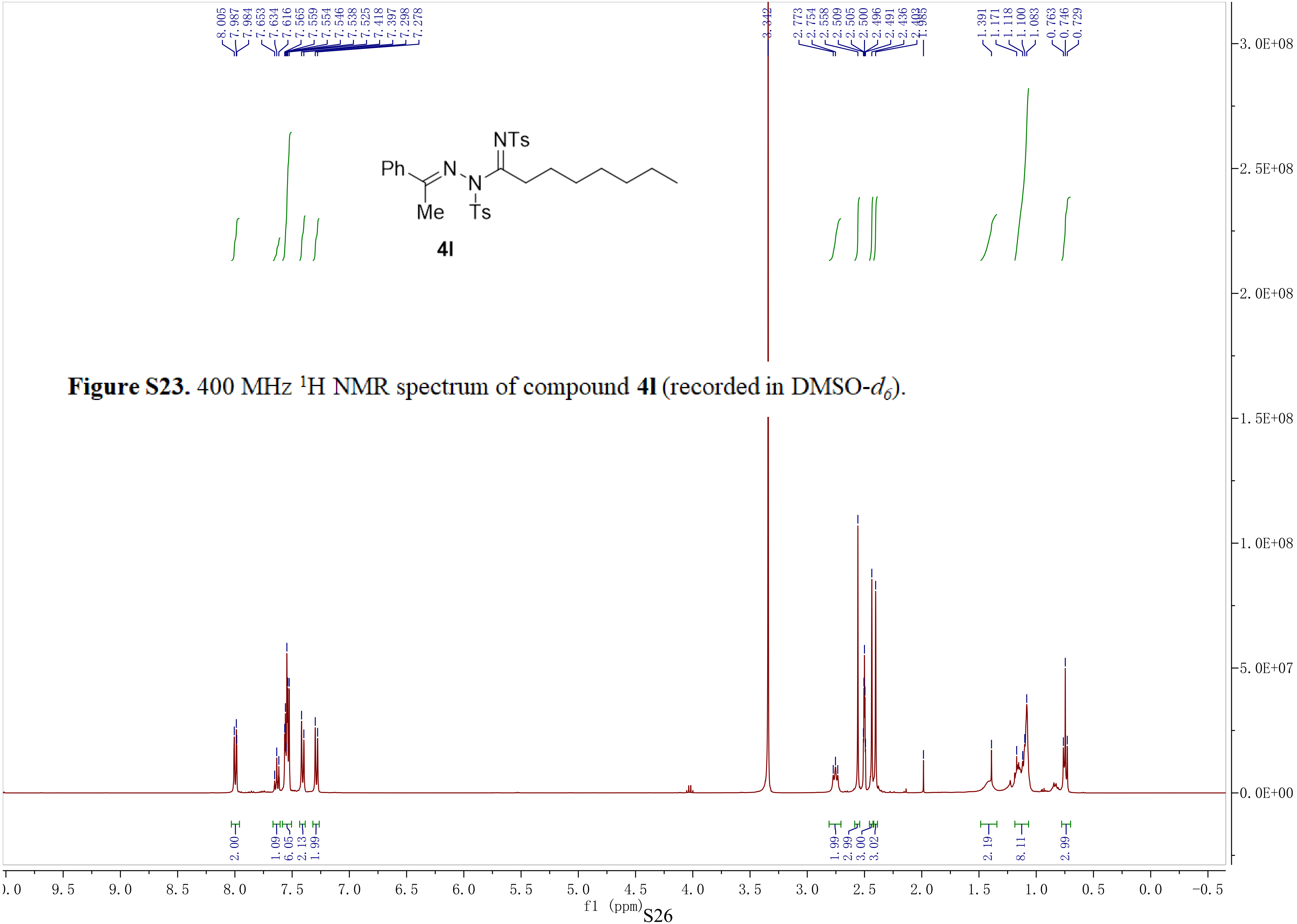

Figure S23. 400 MHz ^{1}H NMR spectrum of compound **4l** (recorded in DMSO-d_6).

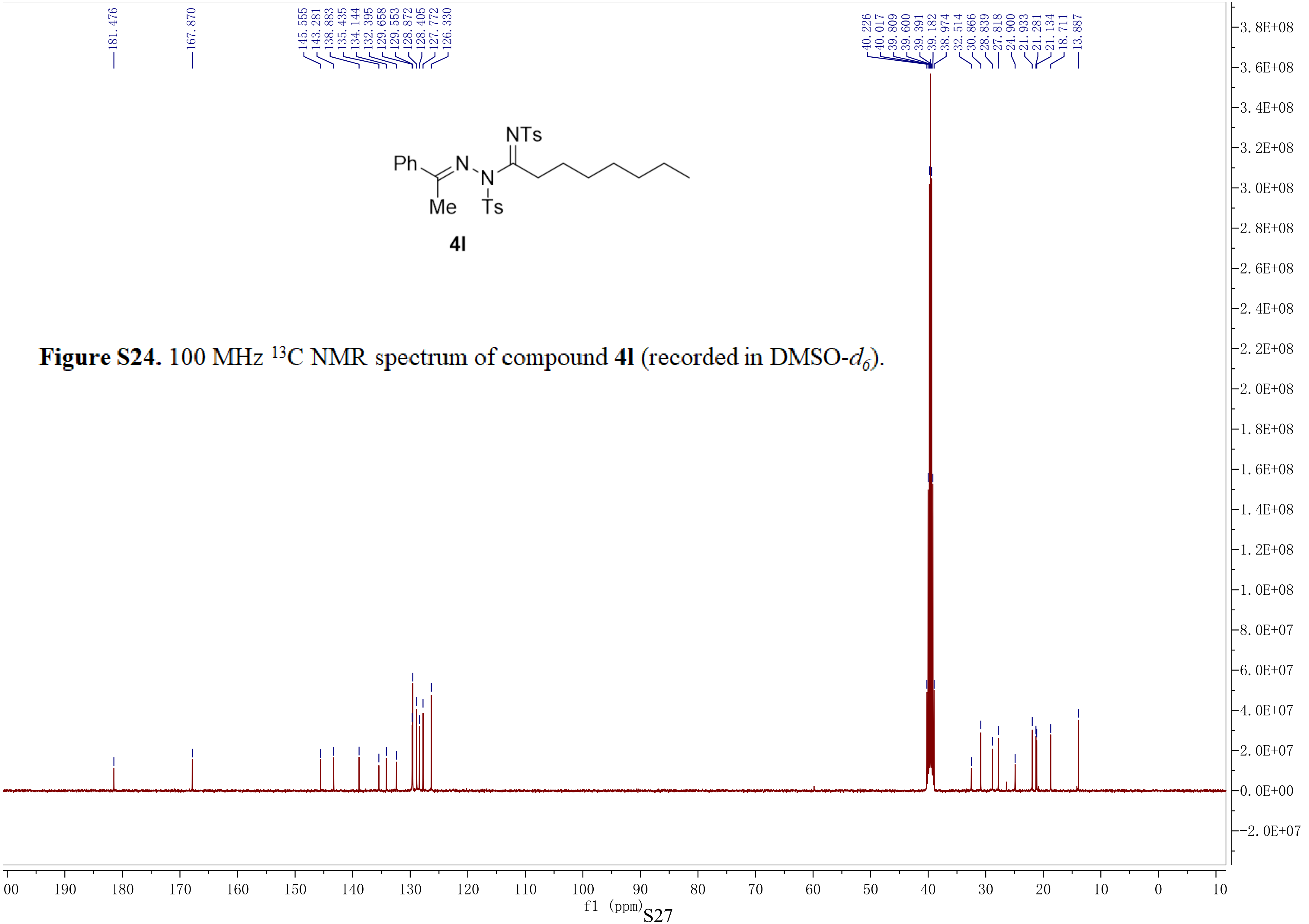

Figure S24. 100 MHz ^{13}C NMR spectrum of compound **4l** (recorded in DMSO-d_6).

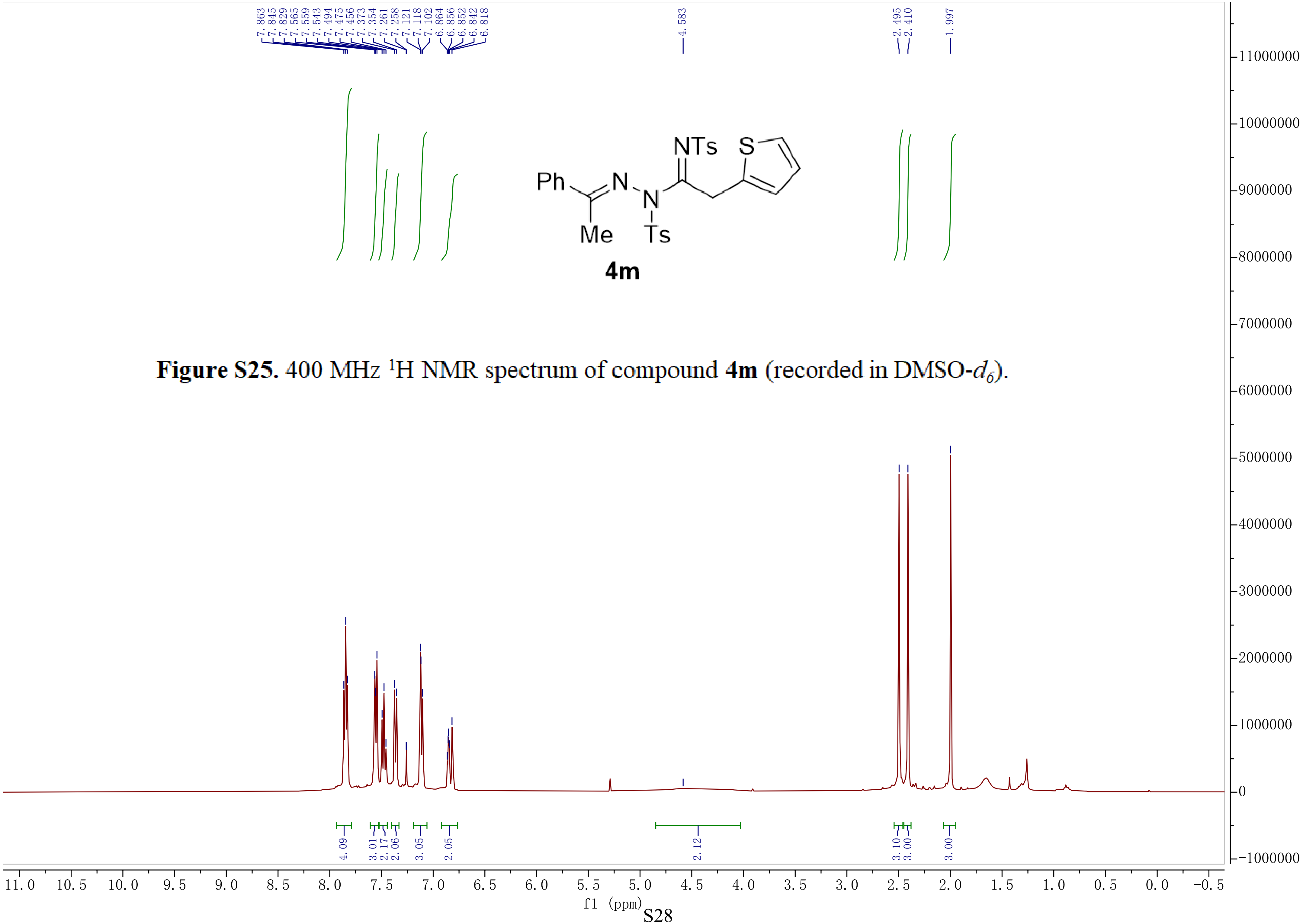

Figure S25. 400 MHz ^{1}H NMR spectrum of compound **4m** (recorded in DMSO-d_6).

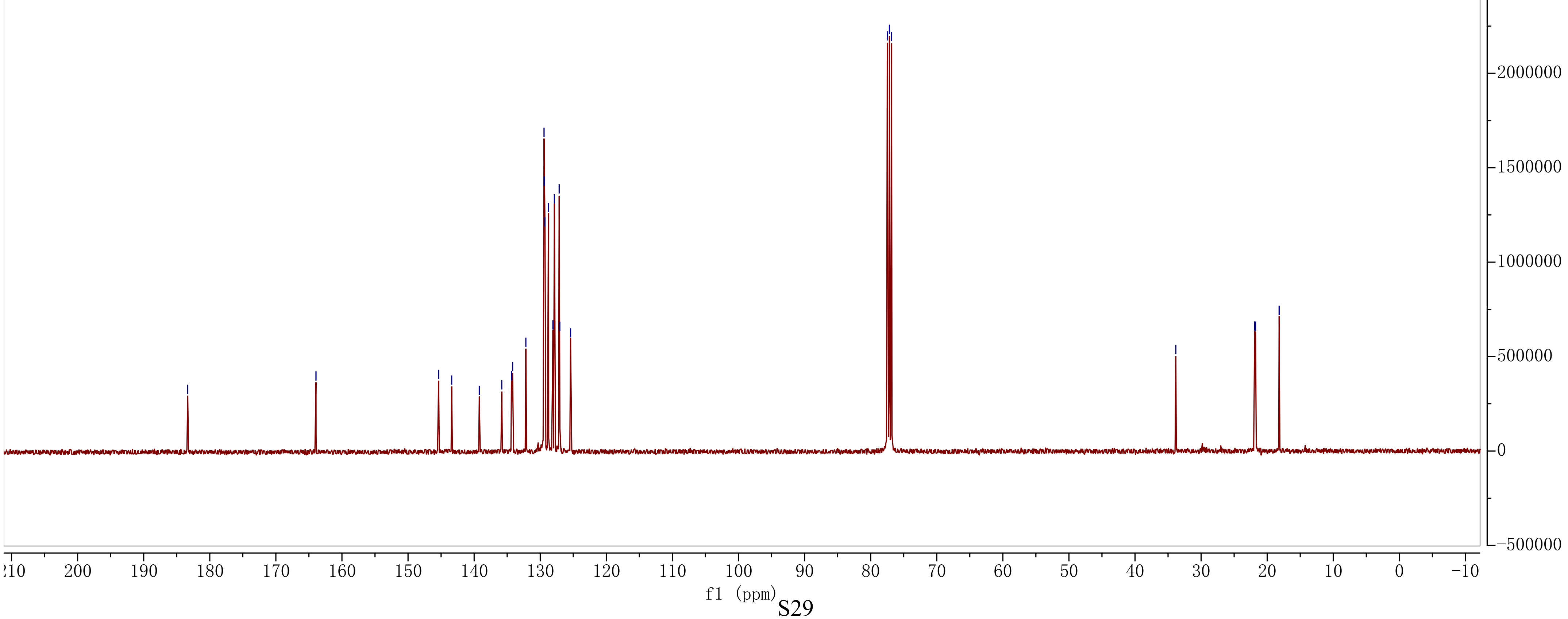

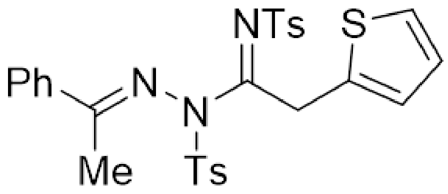

4m

Figure S26. 100 MHz ^{13}C NMR spectrum of compound **4m** (recorded in DMSO-d_6).

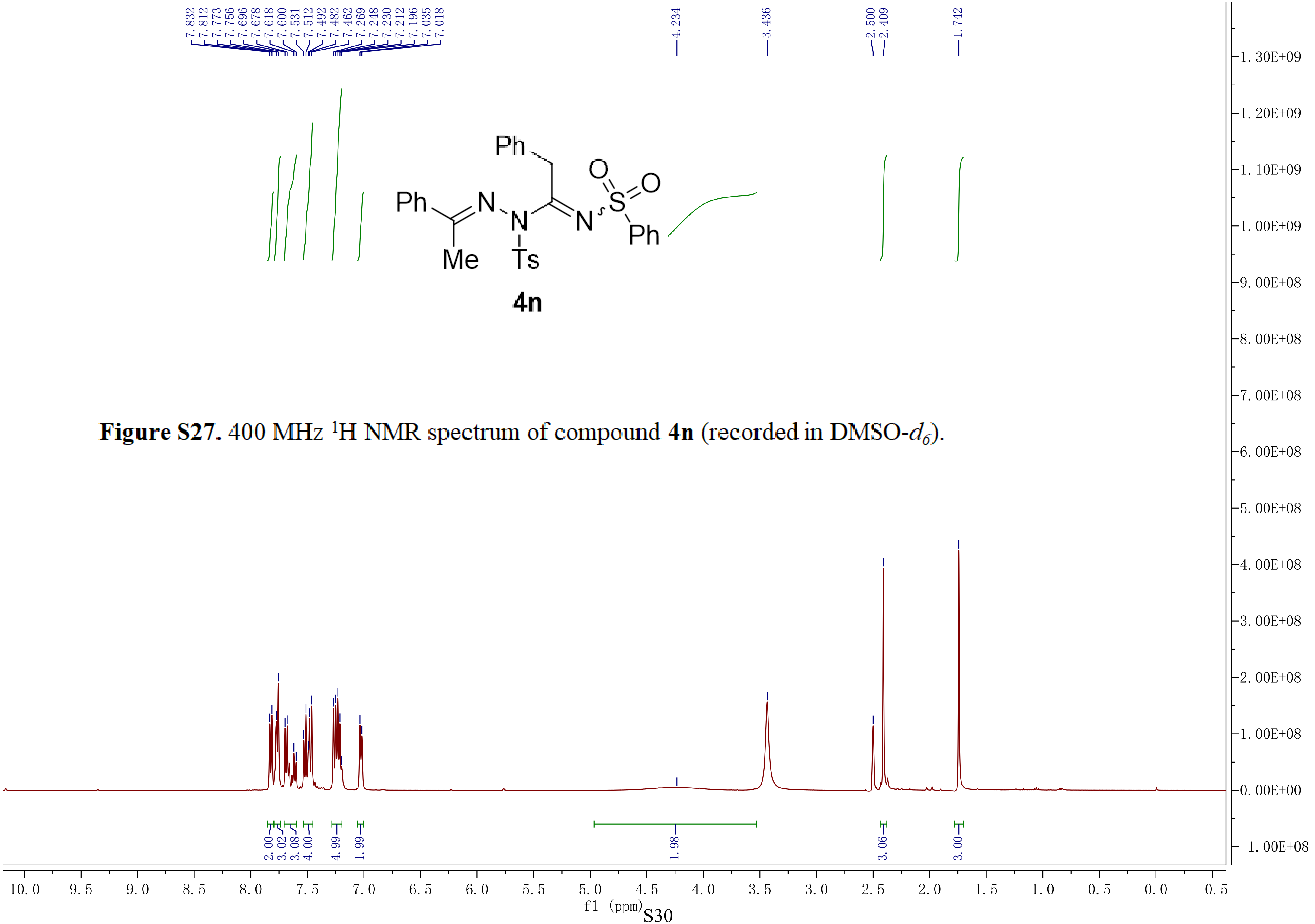

Figure S27. 400 MHz ^{1}H NMR spectrum of compound **4n** (recorded in DMSO-d_6).

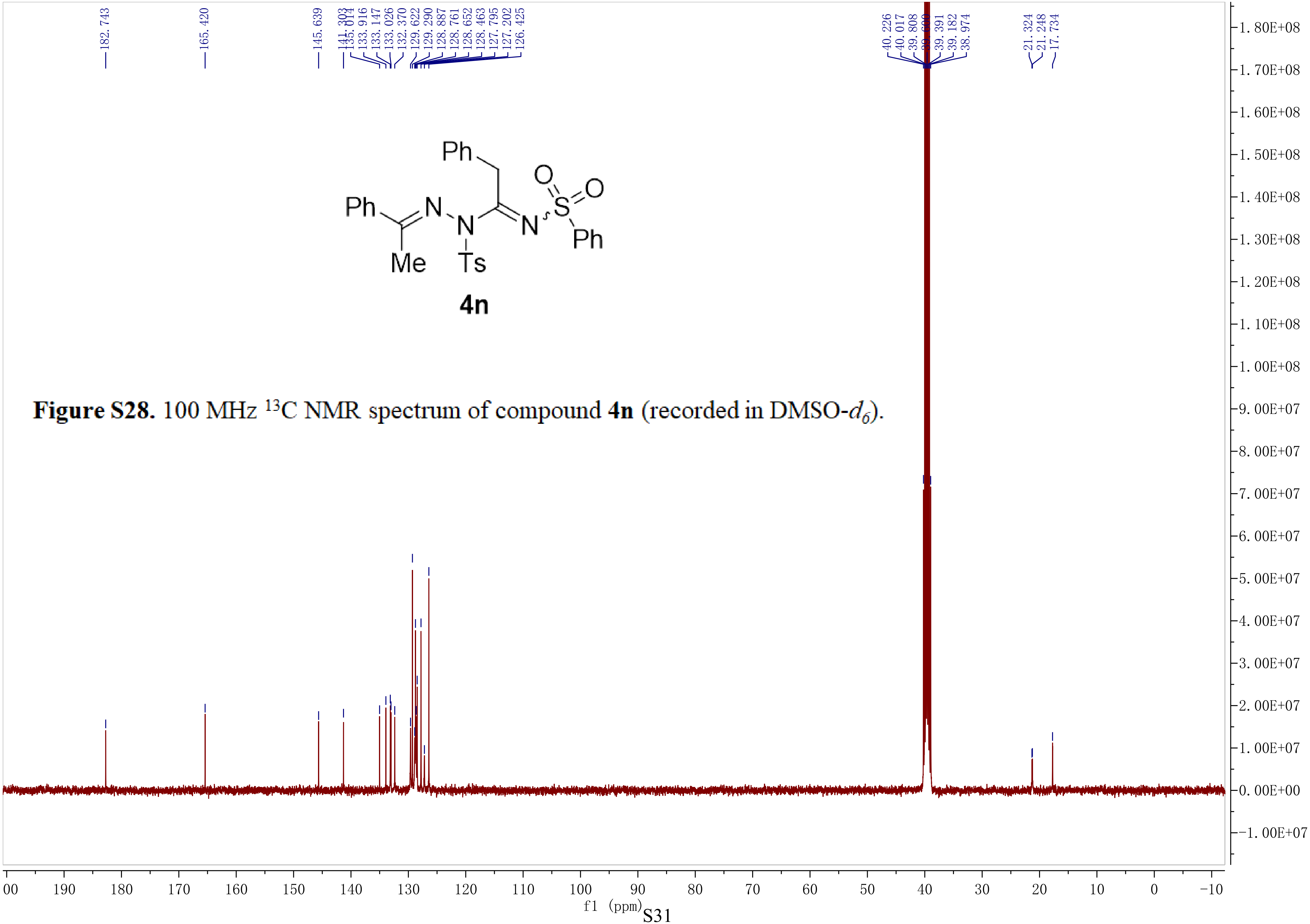

Figure S28. 100 MHz ^{13}C NMR spectrum of compound **4n** (recorded in DMSO-d_6).

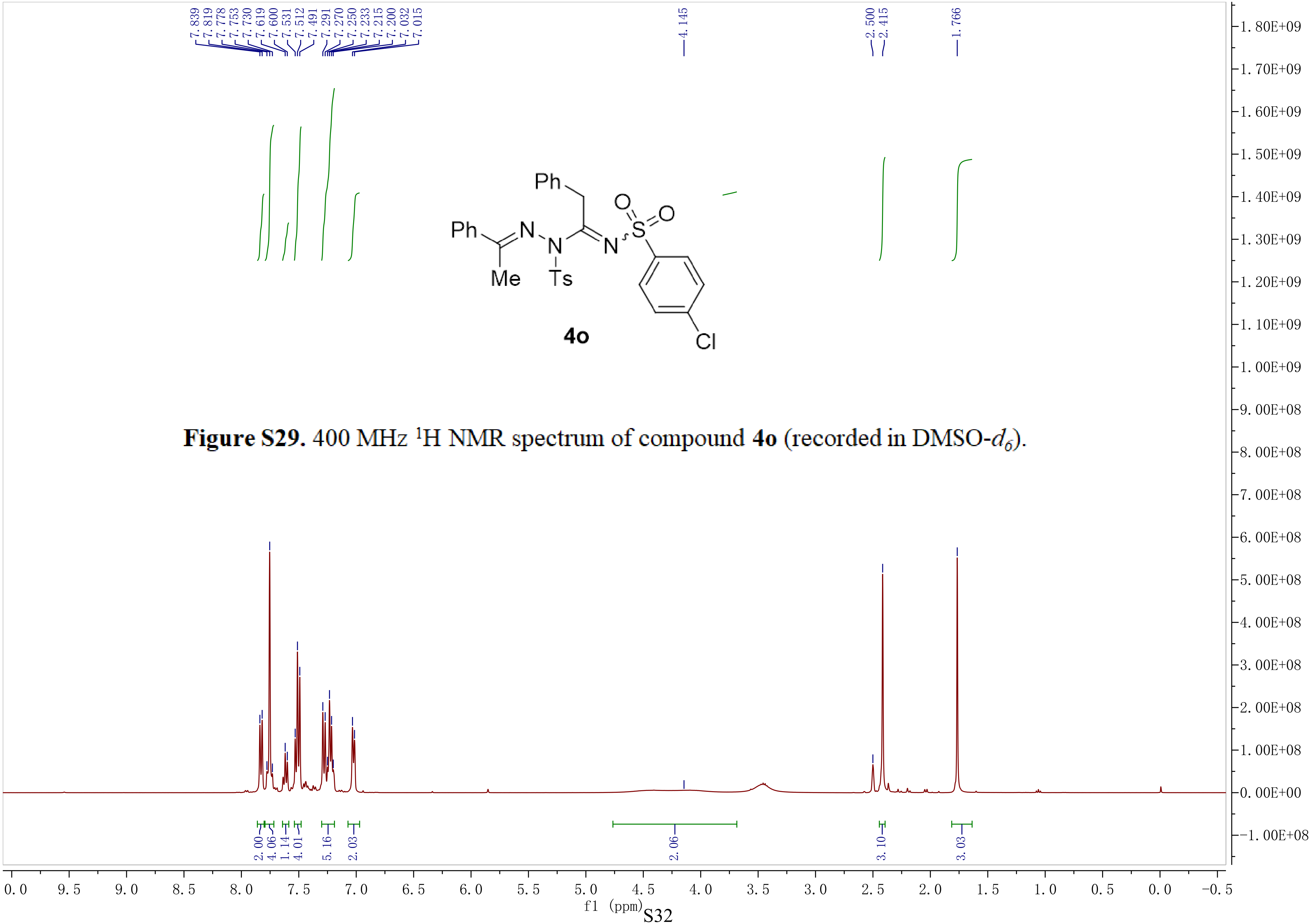

Figure S29. 400 MHz ^{1}H NMR spectrum of compound **4o** (recorded in DMSO-d_6).

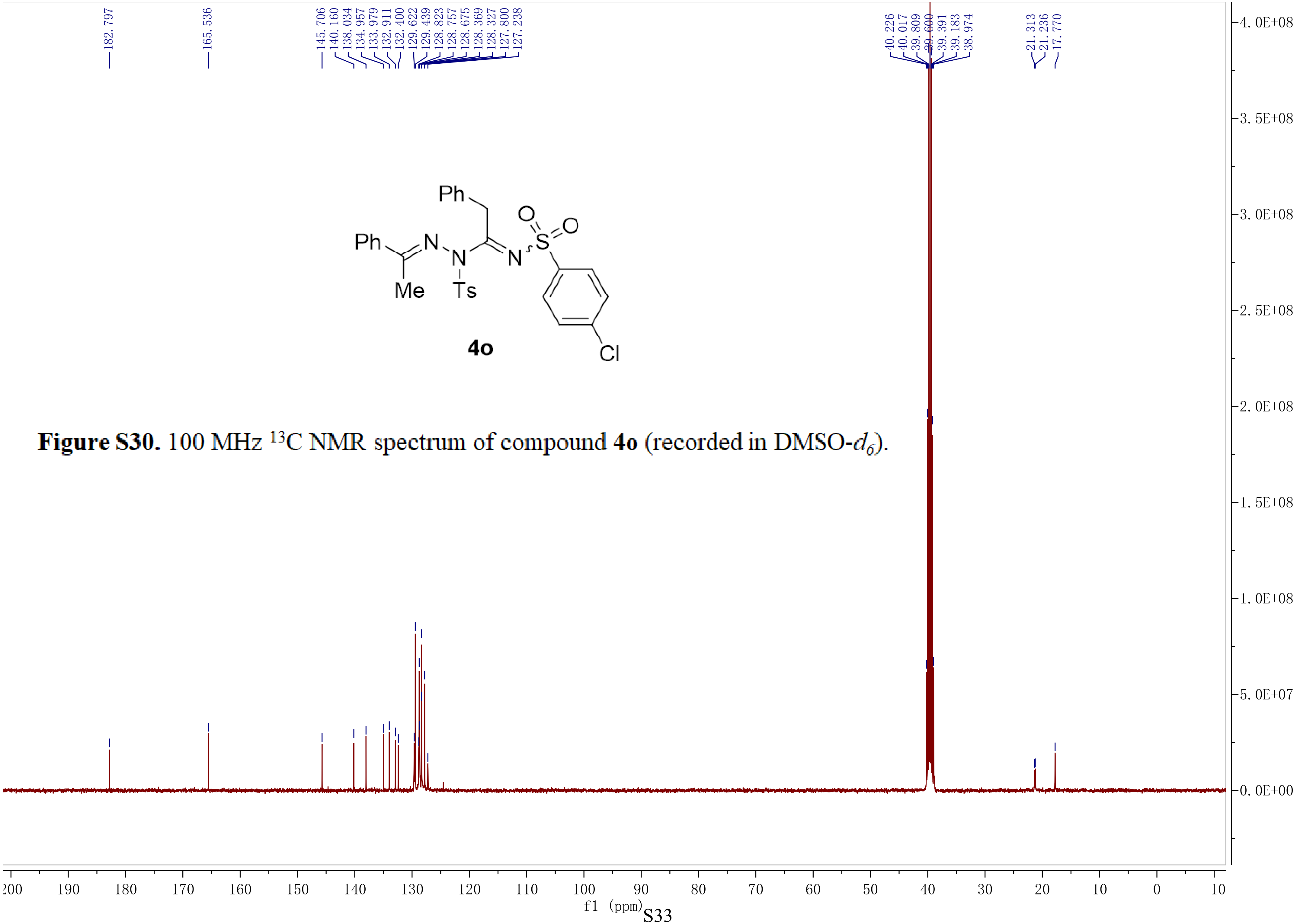

Figure S30. 100 MHz ^{13}C NMR spectrum of compound **4o** (recorded in DMSO-d_6).

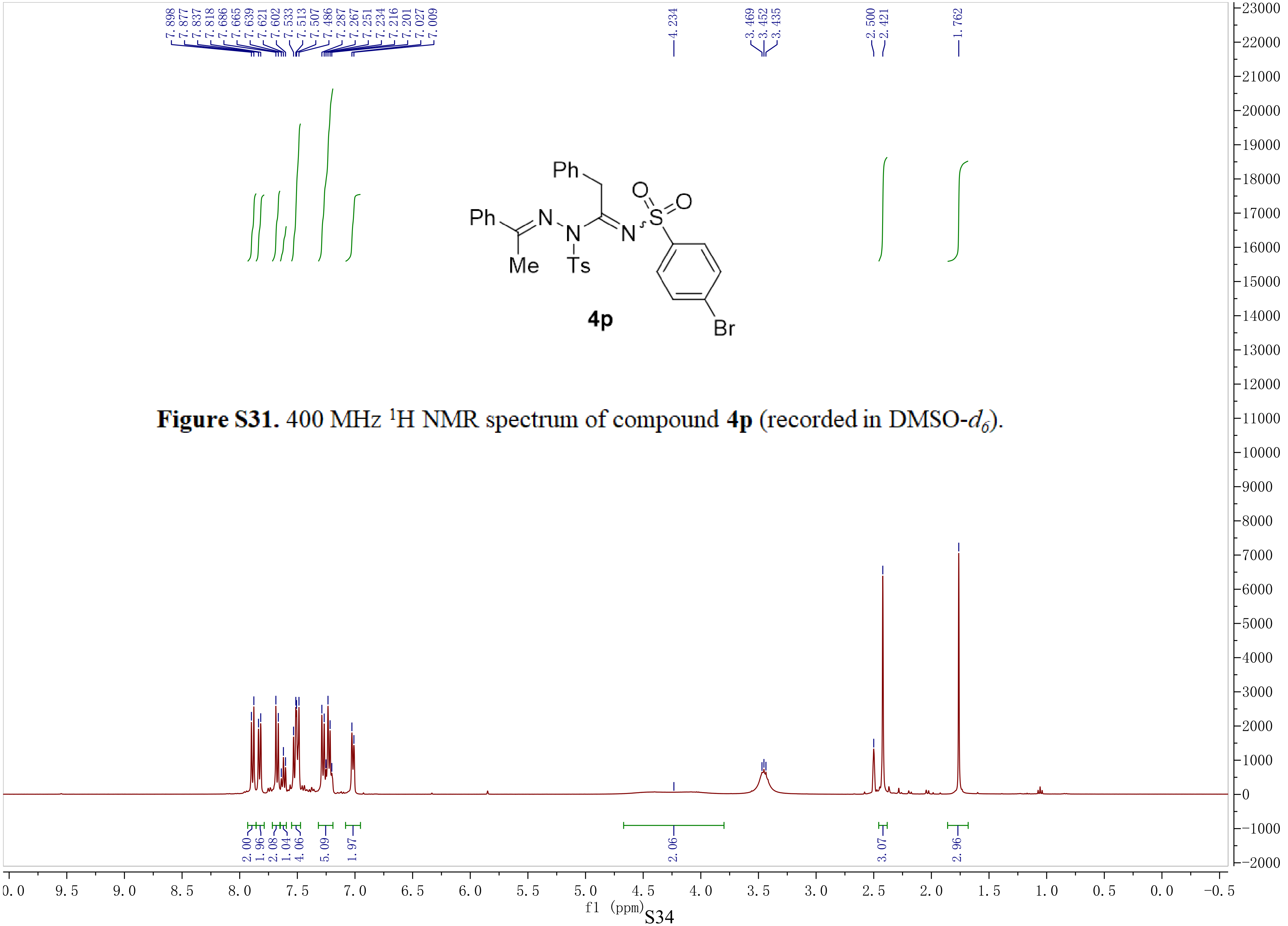

Figure S31. 400 MHz ^{1}H NMR spectrum of compound **4p** (recorded in DMSO-d_6).

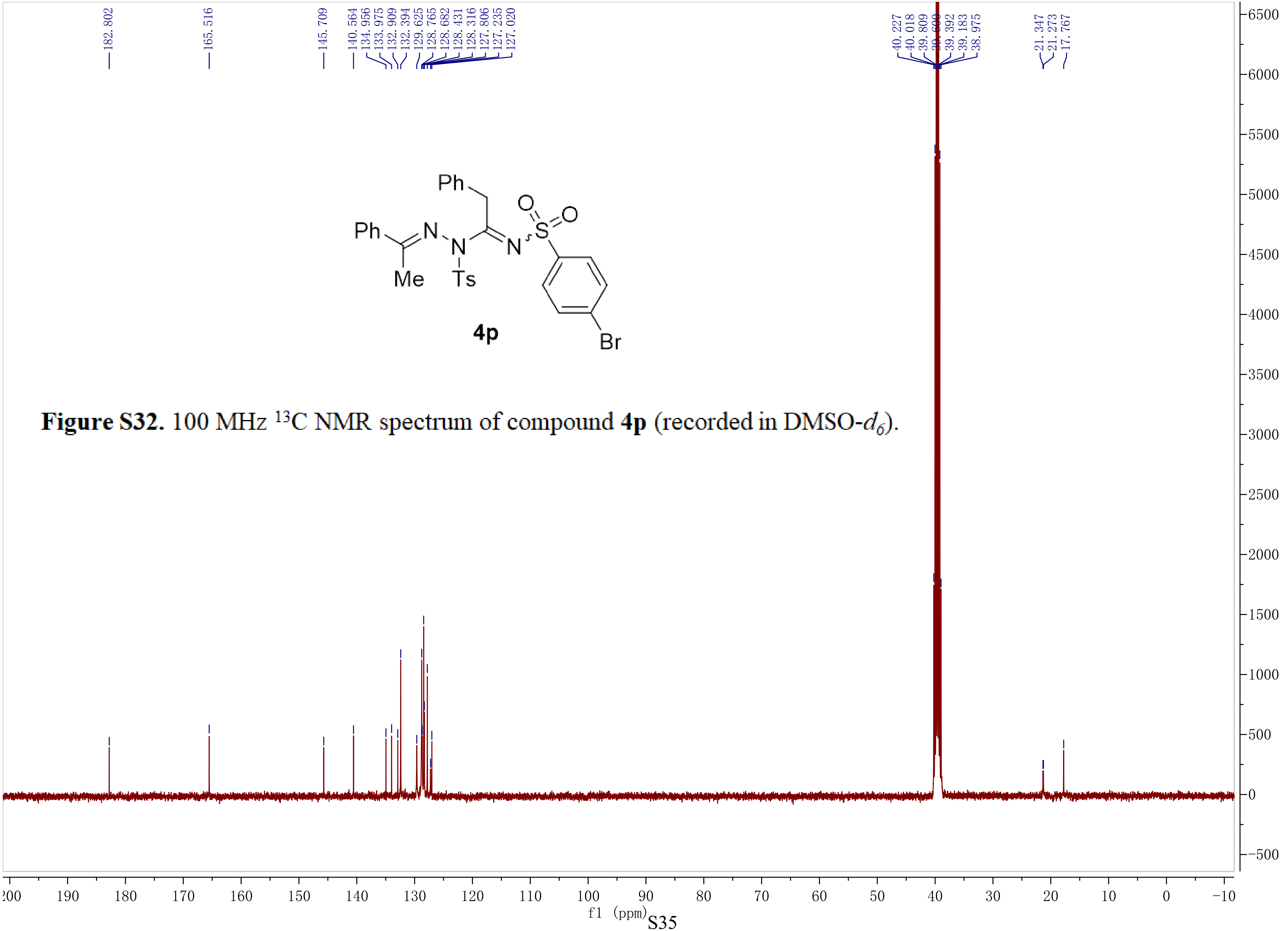

Figure S32. 100 MHz ^{13}C NMR spectrum of compound **4p** (recorded in DMSO-d_6).

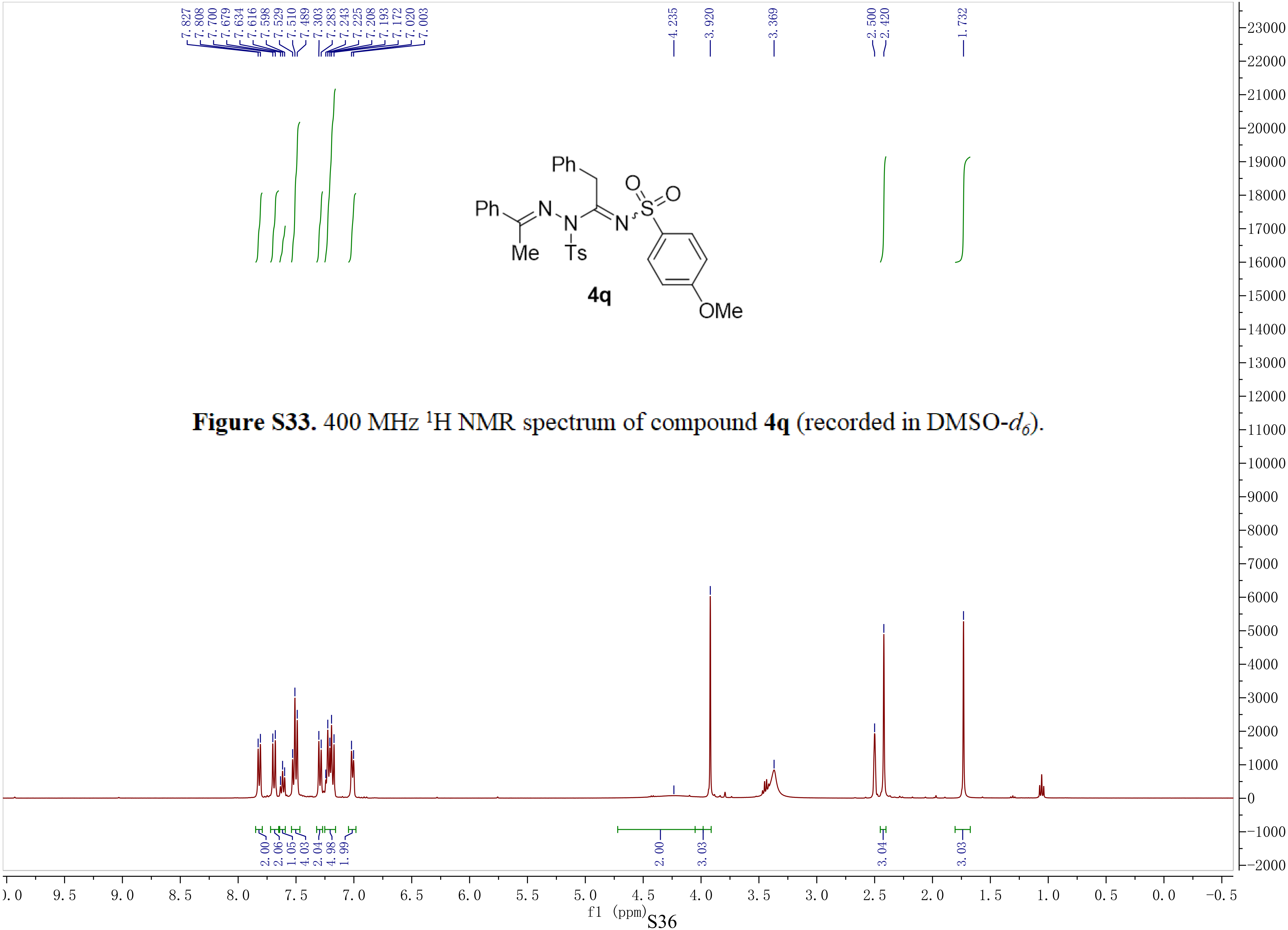

Figure S33. 400 MHz ^{1}H NMR spectrum of compound **4q** (recorded in DMSO-d_6).

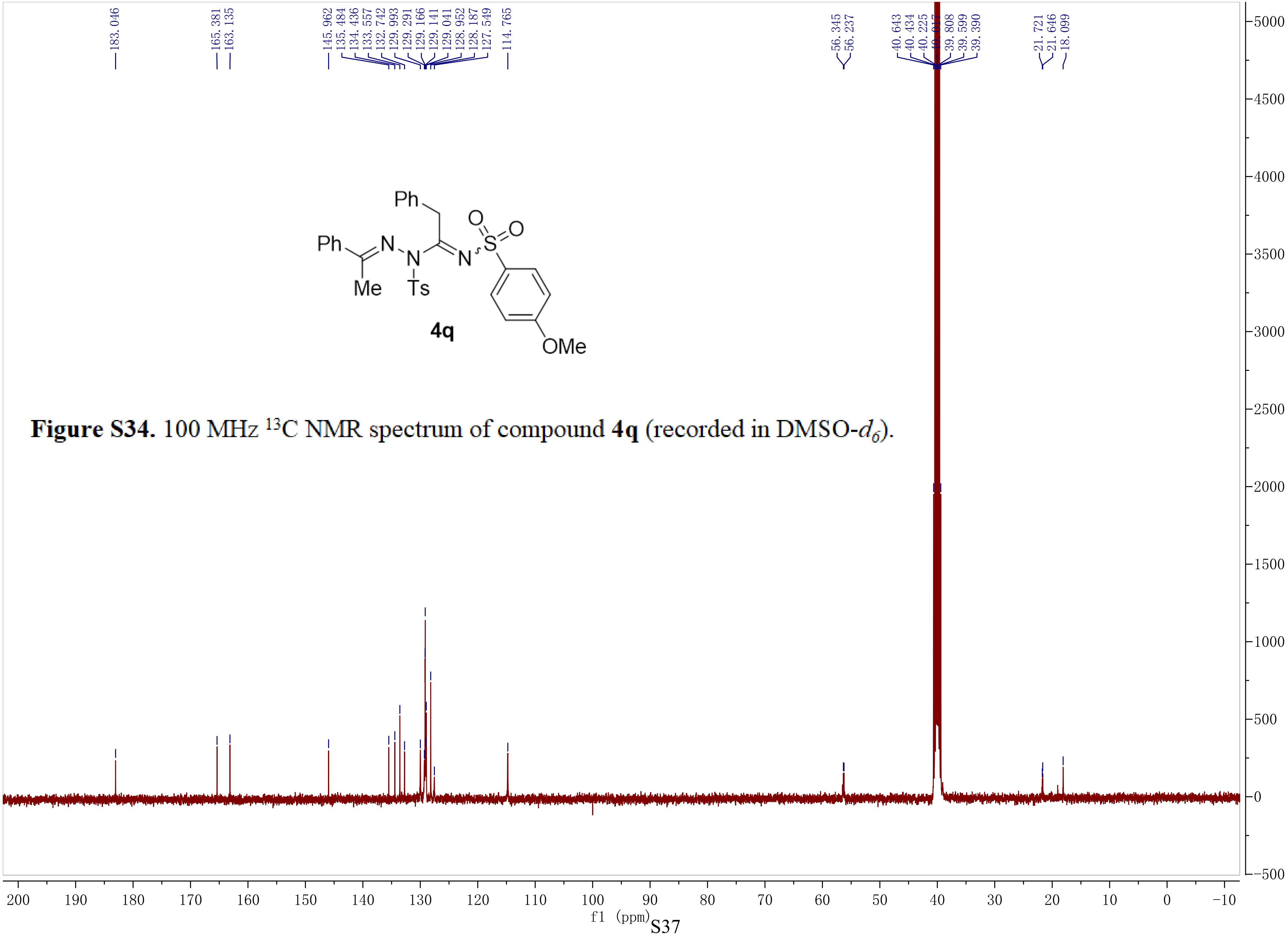

Figure S34. 100 MHz ^{13}C NMR spectrum of compound **4q** (recorded in DMSO-d_6).

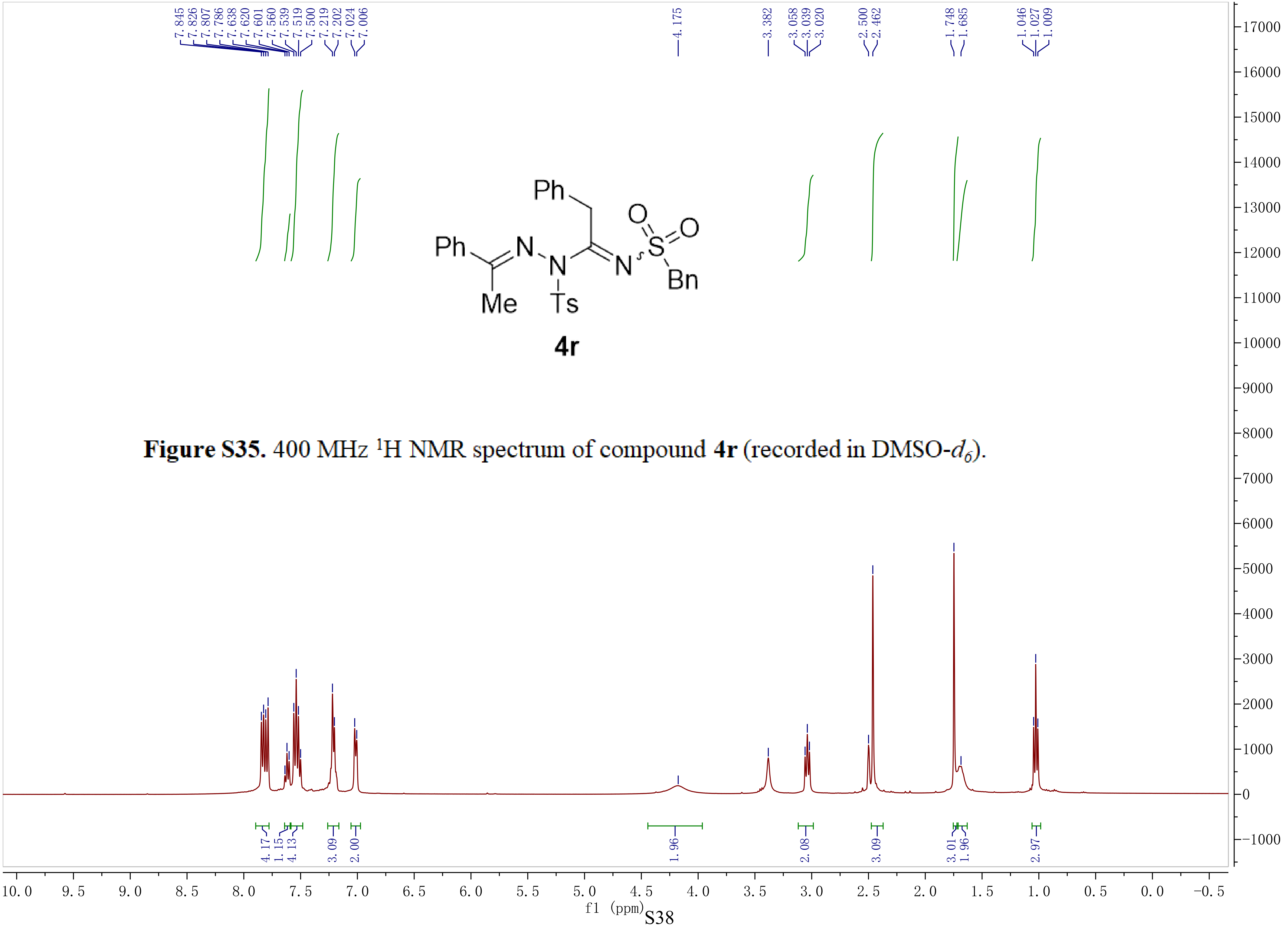

Figure S35. 400 MHz ^{1}H NMR spectrum of compound **4r** (recorded in DMSO-d_6).

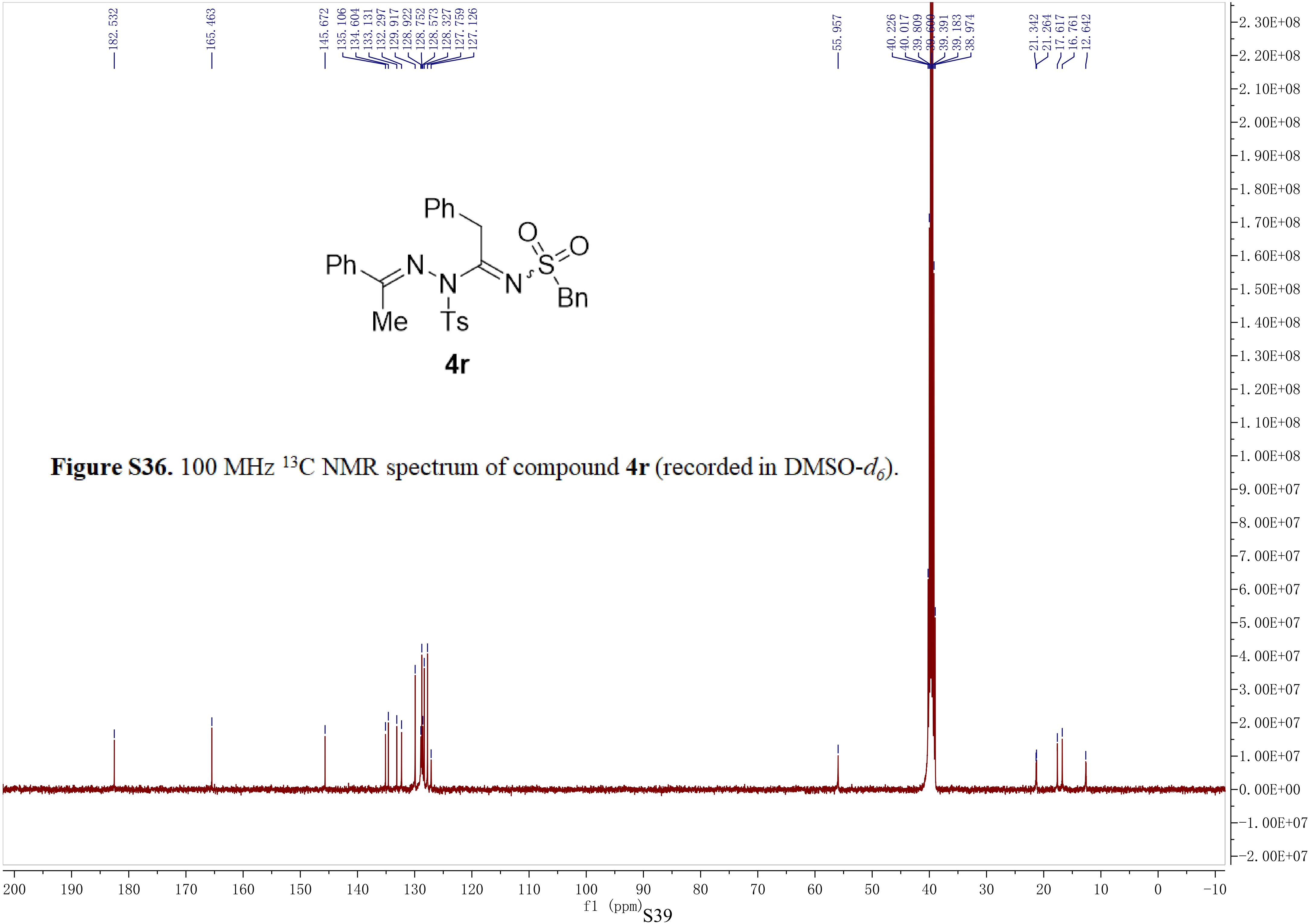

Figure S36. 100 MHz ^{13}C NMR spectrum of compound **4r** (recorded in DMSO-d_6).
